# Purine metabolism regulates *Vibrio splendidus* persistence associated with protein aggresome formation and intracellular tetracycline efflux

**DOI:** 10.3389/fmicb.2023.1127018

**Published:** 2023-03-16

**Authors:** Yanan Li, Thomas K. Wood, Weiwei Zhang, Chenghua Li

**Affiliations:** ^1^State Key Laboratory for Managing Biotic and Chemical Threats to the Quality and Safety of Agro-products, Ningbo University, Ningbo, China; ^2^Laboratory for Marine Fisheries Science and Food Production Processes, Qingdao National Laboratory for Marine Science and Technology, Qingdao, China; ^3^Department of Chemical Engineering, Pennsylvania State University, University Park, PA, United States

**Keywords:** persister cells, purine metabolism, ATP, protein aggresomes, membrane potential, efflux

## Abstract

A small subpopulation of *Vibrio splendidus* AJ01 that was exposed to tetracycline at 10 times the minimal inhibitory concentration (MIC) still survived, named tetracycline-induced persister cells in our previous work. However, the formation mechanisms of persister is largely unknown. Here, we investigated tetracycline-induced AJ01 persister cells by transcriptome analysis and found that the purine metabolism pathway was significantly downregulated, which was consistent with lower levels of ATP, purine, and purine derivatives in our metabolome analysis. Inhibition of the purine metabolism pathway by 6-mercaptopurine (6-MP, inhibits ATP production), increased persister cell formation and accompanied with the decreasing intracellular ATP levels and increasing cells with protein aggresome. On the other hand, the persister cells had reduced intracellular tetracycline concentrations and higher membrane potential after 6-MP treatment. Inhibition of the membrane potential by carbonyl cyanide m-chlorophenyl hydrazone reversed 6-MP-induced persistence and resulted in higher levels of intracellular tetracycline accumulation. Meanwhile, cells with 6-MP treatment increased the membrane potential by dissipating the transmembrane proton pH gradient, which activated efflux to decrease the intracellular tetracycline concentration. Together, our findings show that reduction of purine metabolism regulates AJ01 persistence and is associated with protein aggresome formation and intracellular tetracycline efflux.

## Introduction

When a bacterial infection is treated with antibiotics, even if most of bacteria are susceptible to the antibiotic, there is always a small subpopulation survives, which are called “persisters” (Zhang, 2014; Gollan et al., 2019). Persister cells usually exhibit persistence to multiple antibiotics, low metabolic activity, and high heterogeneity (Khan et al., 2020). Many species utilize the persistence state to permanently colonize their host and establish chronic infections that the immune system and antimicrobial therapies fail to eradicate (Kaldalu et al., 2020; Moldoveanu et al., 2021). Therefore, antibiotic tolerance from persister cells is a threat to human and animal health.

The formation mechanism of cell persistence is complex, different persister cells have specific and complex physiology (Van den Bergh et al., 2017). Previous studies associated persister cell formation mainly focus on cell dormancy mechanism (Lewis, 2007). For example, toxin-antitoxin (TA) systems (Holden and Errington, 2018) the alarmone guanosine tetraphosphate (ppGpp) pathway (Germain et al., 2015) through ribosome hibernation (Wood and Song, 2020), and the SOS response (Dörr et al., 2009) regulate persister cells formation. In addition, *in vivo* studies have shown that *Escherichia coli* causing urinary tract infections in bladder epithelial cells generates biofilms to persist in the face of robust host defenses (Anderson et al., 2003). *Mycobacterium tuberculosis* (Torrey et al., 2016), *Pseudomonas aeruginosa* (Mulcahy et al., 2010), and *Candida albicans* (LaFleur et al., 2010) also use persister cells during infection.

The metabolic state of a bacterium significantly influences its susceptibility to killing by antibiotics (Peng et al., 2015; Jiang et al., 2020; Li et al., 2020), and ATP participates in almost all metabolite synthesis pathways (Zhou et al., 2009; Man et al., 2020). ATP is also one of the best indicators of the energy level of a cell, and it is also the major energy carrier used in cellular metabolism (Hardie et al., 2012; Blundell et al., 2016). Increasing evidences suggest that the variation in the level of ATP serves as a major mechanism of persister cells formation (Cheng et al., 2014; Parry et al., 2014; Shan et al., 2017), for most of the antibiotic targets require ATP to function, when ATP is reduced or depleted, antibiotic targets are inactivated and cells dormant (Shan et al., 2017). ATP depletion appears to be an effective mechanism for *Staphylococcus aureus* and *E. coli* persister cells formation (Cheng et al., 2014; Conlon et al., 2016). However, how ATP is depleted and how ATP-depletion are linked to persister cells formation remain unclear.

There are reports that the decreased ATP reduces the rate of protein synthesis, chaperone activity and growth when an organism experiences slow-growth conditions (Watson et al., 1998; Manning, 2013; Pu et al., 2018). It is important to point out that ATP depletion in starved *E. coli* cells causes protein aggresome formation, which depends on the dynamics of ATP to regulate the depth of dormancy of bacteria, thereby affecting the level of antibiotic resistance (Pu et al., 2018). In the yeast stress adaption model, nutrient scarcity causes cellular ATP depletion and a drop in cytosolic pH, which in turn promotes macromolecule granule formation and cytoplasmic solidification, thereby enhancing cell dormancy (Munder et al., 2016). Meanwhile, Pu et al. (2018) reported that in nutrient-depleted cells, aggresome formation accompanies intracellular pH drop in *E. coli* persisters. These studies indicate a correlation between protein aggresome formation and dormancy. In addition, lower ATP levels result in reduced synthesis of proteins, DNA, peptidoglycan, decreases protein solubility, and quality control (Patel et al., 2017; Wilmaerts et al., 2019), which are also closely related to cell dormancy and persister cell formation.

Antibiotics tend to achieve a lower intracellular concentration in *E. coli* persistence cell, indicating either lower rate of drug uptake or higher rate of drug efflux, or a combination of both (Alekshun and Levy, 2007; Pages et al., 2008; Pu et al., 2016, 2017; Lu et al., 2021). The antibiotic accumulation of gram-negative bacteria is mainly affected by two factors of membrane permeability and efflux activity (Pu et al., 2016). Previous studies have reported the formation of *E. coli* persister cells were associated with the reduction in membrane potential (Verstraeten et al., 2015; Roy et al., 2021). With 5 mM salicylate treatment could collapse the membrane potential through the production of ROS, thereby inducing *E. coli* persistence (Wang et al., 2017). Inversely, bacterial multi-drug efflux systems actively pump antibiotics out to reduce cellular drug accumulation, thus facilitating bacterial survival (Sun et al., 2014; Pu et al., 2016; Griffith et al., 2019; Byrd et al., 2021; Le et al., 2021). Interestingly, these efflux pumps are powered by membrane potential and transmembrane pH gradient across the bacterial cell membrane (Paulsen et al., 1996). There is an important regulatory mechanism between membrane potential and efflux pump in *E. coli* persister cells (Byrd et al., 2021), membrane potential can drive both the influx of aminoglycosides and the pump of antibiotics out of the cell (Taber et al., 1987; Paulsen et al., 1996), but this regulation of other bacteria is unknown.

*Vibrio splendidus* AJ01 is a gram-negative bacterium and can infect a broad host range of cultured animals in marine environments, resulting in various diseases and high mortality, thus leading to high economic losses in aquaculture. Previously, we found that 10 × MIC tetracycline could induce the formation of AJ01 persisters which had low metabolism (Li et al., 2021). Here, we performed integrated transcriptome and metabolome analysis between AJ01 persister cells and active cells and found that an imbalance in purine metabolism was associated with AJ01 persister cells formation, accompanied by low intracellular ATP levels and the formation of protein aggresome. Meanwhile, the decrease in purine metabolism also affected membrane potential and efflux, reducing intracellular accumulation of tetracycline. These results provide new insights into understanding AJ01 persister cell formation was regulated purine metabolism.

## Materials and methods

### Transcriptome and metabolome analysis

Bacterial strains *V. splendidus* AJ01 used in this study were previously isolated from SUS diseased sea cucumber and characterized in our laboratory (Zhang et al., 2016). Bacterial were cultured at 28°C in 2216E medium [5 g/L tryptone, 1 g/L yeast extract, and 0.01 g/L FePO_4_ in filtered natural seawater]. The overnight bacteria culture was diluted 1:100 into fresh 2216E medium and cultured until it reached at different growth stages (OD_600_ = 0.6, 1.2, and 2.0. These three OD’s are represented log phase, early stationary phase, and late stationary phase, respectively). Tetracycline-induced persister cells were selected as described by our previous work (Li et al., 2021). The samples without tetracycline treatment at OD_600_ of 0.6, 1.2, and 2.0 were served as the control. RNA isolation, cDNA library preparation, Illumina sequencing, and mapping of reads were conducted on the Illumina HiSeq/MiSeq sequencing platform at Novogene Biotech (Beijing, China). The trimming and quality control of raw Illumina reads were conducted to obtain clean reads. The clean reads were mapped to the genome of *Vibrio atlanticus* strain LGP32 (FM954973.2) using Bowtie2 (Langmead and Salzberg, 2012). Cluster Profiler software was used to analyze KEGG pathway enrichment of differential gene sets. *p*_adj_ < 0.05 was considered as the threshold of significant enrichment. *p*_adj_ was the corrected value of *p*, and a small *p*_adj_ value of differentially expressed gene indicated high significance of the differential expression (Jin and Hammell, 2018). *p*_adj_ = p^*^ *m*/*n*, such that *p* is the original value of *p*, *m* is the maximum number of tests that minimizes Padj, and *n* is the total number of tests (Hu et al., 2018). Interactive Pathways (iPath) analysis using iPath3.0.^1^

Metabolome samples preparation were the same as the transcriptome as mentioned above. The overnight bacteria culture was diluted 1:100 into fresh 2216E medium and cultured until it reached at OD_600_ = 1.2. The sample numbers of *VS* and VST were labeled. Each sample had six biological replicates. The resulting samples were used for non-target metabolomics liquid chromatography-mass spectroscopy analysis and conducted at Shanghai Luming Biological Technology Co. Ltd (Shanghai, China). Multivariate statistical analyses were applied for metabolites. Normalization and analysis of metabolome data were completed using Microsoft Excel. Principal-component analysis (PCA) was used to reduce the high dimension of the data set and analyze the covariance variation and emphasize the outlier in clustering. The significantly altered abundance of metabolites was analyzed and outlined in KEGG (https://www.kegg.jp/).

### Antibiotics and inhibitors assay

Five hundred μM 6-MP (Krishnan et al., 2009) and 50 μM mycophenolic acid (MPA) (Zhou et al., 2020) were used to inhibit purine metabolism to ATP and GTP, respectively. For dose-dependent inhibition assay, 1 mL overnight cultured *V. splendidus* AJ01 was treated with three concentrations of 6-MP (50 μM, 250 μM, and 500 μM) and four concentrations of MPA (5 μM, 10 μM, 20 μM, and 50 μM) for 3 h before adding 250 μg/mL tetracycline for 4 h at 28°C, respectively. Then the cells were collected by centrifugation at 5,000 g for 5 min. The cells were resuspended and washed with phosphate buffered saline (PBS) [137 mM NaCl, 2.7 mM KCl, 8 mM Na_2_HPO_4_, and 1.5 mM KH_2_PO_4_] three times to isolate unlysed persister cells and total cells. One milliliter of the cell culture was serially diluted by PBS, and 10 μL samples were spotted onto 2216E agar plates. Colonies were counted after overnight incubation at 28°C, and those dilutions yielding 20–200 colonies were enumerated to calculate colony-forming units (CFU). For membrane potential inhibition, 10 μM cyanide m-chlorophenyl hydrazone (CCCP) was added to the bacteria for 30 min before tetracycline treatment. Inhibition of efflux pump was treated with 100 μM NMP was added to the bacterial culture medium at the same time points with tetracycline.

### Measurement of intracellular ATP

Intracellular ATP levels of the bacteria cultures were measured using BacTiter-Glo Microbial Cell Viability Assay (Promega, G8231) following the manufacturer’s instructions. Cells were suspended to an OD_600_ of 0.6 (around 10^5^ bacterial cells) in 2216E medium incubated with the addition of various concentration gradient of chemicals before ATP measurement under a fluorescence microplate reader (ThermoFisher). Intracellular ATP concentration was determined through normalizing ATP levels by cell number and single cell volume.

### Insoluble protein isolation and mass spectrometry

One milliliter overnight cultured *V. splendidus* AJ01 was treated with 6-MP or MPA for 3 h before adding 250 μg/mL tetracycline for 4 h at 28°C, respectively. And 1 mL overnight cultured cells were treated with exogenous ATP and 250 μg/mL tetracycline for 4 h at 28°C. For cells staining, cells were collected by centrifugation at 5,000 g for 5 min, resuspend and washed with PBS three times to isolate unlysed persister cells and total cells. Then cells were incubated with 1 μM fluorescein isothiocyanate (FITC) (Sigma, aldrich) for 20 min (Polak et al., 2019). Three washing steps with 1 mL PBS were carried out to remove the free fluorescent dye (Polak et al., 2019). For protein aggresomes visualization, cellular “dark foci” (brightfield) and “green fluorescent foci” (green fluorescent staining) as a marker for the formation of protein aggresomes (Pu et al., 2018). Intracellular green fluorescent foci were imaged using FITC filter sets. Image analysis software and counted dark foci with the ImageJ (Le et al., 2021).

The method for insoluble protein aggresomes isolation was described by Pu et al. (2018). Two milliliters of bacterial cultures at OD_600_ = 1 were rapidly cooled on ice for 10 min and centrifugated at 5,000 g for 10 min at 4°C to collect bacteria. Pellets were suspended in 100 μL buffer A [10 mM potassium phosphate buffer (PPB), pH 6.5, 1 mM EDTA, 20% (w/v) sucrose, 1 mg/mL lysozyme] and incubated for 30 min on ice. Cell lysate was added with 500 μL of buffer B (10 mM PPB, pH 6.5, 1 mM EDTA), then sonication in ice bath. Intact cells were removed by centrifugation at 2,000 g for 10 min at 4°C. The pellet fractions were resuspended in 500 μL of buffer C (buffer B with 2% NP40) to dissolve membrane proteins and the aggregated proteins were isolated by centrifugation at 2,000 g for 10 min at 4°C. Then the membrane and aggregated proteins were centrifugation by 15,000 g for 30 min at 4°C. The pellet fractions were resuspended in 400 μL of buffer B by brief sonification and centrifugation by 15,000 g for 30 min at 4°C. NP40-insoluble pellets were washed twice with 400 μL of buffer B and re-suspended in 50 μL of buffer B. Aggregated proteins of each sample tetracycline-induced persister cells and active cells were cut out of the gel and characterized on the LC–MS platform at a biotechnology company (Shanghai Sangon Biological Engineering Technology & Services Co. Ltd., China). VENNY 2.1^2^ analyzed the common of protein aggresomes. KOBAS 3.0^3^ analyzed the enrichment of KEGG.

### Measurement of intracellular tetracycline concentration

Intracellular tetracycline concentrations of the cultures were measured using Tetracycline enzyme-linked immunosorbent assay (ELISA) diagnostic kit (Lunchangshuo, SU-B99855) following the manufacturer’s instructions. The cells were collected by centrifugation at 5,000 g for 5 min and washed three times with PBS. An aliquot of 5 mL was sonicated (30% output, 2 s ultrasound, and 8 s interval time for a total of 2 min) in an ice bath. The resulting supernatant was collected for the detection of tetracycline following the instructions of the tetracycline ELISA diagnostic kit. Intracellular tetracycline concentrations were measured under a microplate reader (Molecular Devices, SpectraMax 190) at 450 nm.

### Measurement of membrane potential by DiOC_2_(3) fluorescence

Membrane potential of cells was measured using BacLight™ Bacterial Membrane Potential Kit (Life Technologies, B34950) following the manufacturer’s instructions. One milliliter of 10^6^ CFU of bacteria were used and stained with 10 μL of 3 mM 3, 3-diethyloxa-carbocyanine iodide [DiOC_2_(3)], followed by incubation for 30 min. Cultures with the addition of various concentration gradient of CCCP (0 μM, 10 μM, 25 μM, and 75 μM) to the depolarized sample and mix for 30 min. Samples were analyzed using a FACScan flow cytometer (Becton Dickinson, San Jose, CA) using the following settings: fluorescein isothiocyanate (FITC) voltage, 250 V; mCherry voltage, 650 V. The red/green (mCherry/FITC) values for each cell were determined, normalized, and then compared between samples. The relative membrane potential of test samples was determined.

To observe the single cell was performed on an agarose gel pads as described previously (Zhang et al., 2019). Low melting agarose (1.5%) were prepared using the sandwich method (Kim and Gadd, 2019). Briefly, 1.5% of low-melting agarose was added to 2216E liquid medium and melted by microwaving. DiOC_2_(3) exhibits green fluorescence in all bacterial cells, but the fluorescence shifts toward red emission as the dye molecules self-associate at the higher cytosolic concentrations caused by larger membrane potentials. When we observed single cell membrane potential changes using fluorescence microscopy, in cells with higher cytoplasmic concentrations, self-associating red fluorescence still appears, however only for 1–2 s. Subsequently, the red fluorescence of bacteria at high membrane potential disappeared, and the green fluorescence of bacteria with high membrane potential is stronger than that of low membrane potential. Therefore, the DiOC_2_(3) fluorescence value was measured under a fluorescence microscope (ZEISS, Axiovert A1) maintained at 28°C with the excitation wavelength at 488 nm and detected with emission filters suitable for FITC (green).

### Measurement of tetracycline efflux by Hoechst 33342 fluorescence microscopy

To make fluorescence measurements, cells were treated with 0.1 μM Hoechst 33342 for 60 min at 28°C in the dark. For the efflux pump inhibition experiment, bacteria cultures were added N-Methylpyrrolidone (NMP) incubated at 28°C for 4 h, which can effectively block efflux pumps through competitive substrate export (Donner et al., 2017; Camp et al., 2021). The cells then were loaded onto 1.5% agarose pads, which made with the same 2216E growth medium. Cells were imaged with a fluorescence microscope. Intracellular Hoechst 33342 were imaged using 4′,6-diamidino-2-phenylindole (DAPI) filter sets. Image Analysis Software and analyzed with the ImageJ.

### Intracellular pH measurement

Intracellular pH measurement was conducted as previously described (Song et al., 2021). Briefly, the tested bacteria were washed with PBS for three times and were then treated with 10 μM BCECF-AM (Beyotime, S1006) for 30 min at 28°C. The fluorescence intensity value was measured under a fluorescence microscope with the excitation wavelength at 488 nm and emission wavelength at 535 nm.

### qRT-PCR

Quantitative real-time PCR (qRT-PCR) was carried out as described previously (Li et al., 2021). Total RNA was isolated from cells using a Bacterial RNA Isolation kit (Omega, R6950-01). The purity and quantity of the total RNA were determined using a NanoDropTM 2000 spectrophotometer (Thermo Fisher Scientific). By using a Primescript™ 1st cDNA Synthesis kit (TaKaRa, RR036A) reverse transcription-PCR was carried out on 1 μg of total RNA according to the manufacturer’s instructions. qRT-PCR was performed in 96-well plates, amplifications were performed in a 20 μL of reaction volume containing 8 μL of diluted cDNA (1:100 dilution of cDNA with sterile water), 0.8 μL of each primer, and 10.4 μL of SYBR Green Mix (Takara, DRR420A). The reaction mixtures were carried out in an ABI 7500 RT-PCR detection system (Applied Biosystems). The 2^-△△CT^ method was used to analyze the expression level of each gene. Data are shown as the relative mRNA expression compared with control with the endogenous reference 16S rRNA gene. Primers used for qRT-PCR are listed in the Supplementary Table S1.

## Results

### Integrated transcriptome and metabolome analysis reveals purine metabolism is associated with persister cells formation

In our previous study, we defined the live AJ01 by 10 × MIC tetracycline treatment for 4 h as tetracycline-induced AJ01 persister cells (Li et al., 2021), which was consistent with Keren ‘s experimental method (Keren et al., 2011). To investigate which pathways are associated with tetracycline-induced persister cell formation, integrated transcriptomes and metabolomes were employed to identify the differentially expressed genes (DEGs) and metabolic products of tetracycline-induced persister cells (VST) compared to active cells (*VS*). The DEGs of VST vs. *VS* at three stages were determined (Supplementary Figure S1A). A total of 1,166 DEGs containing 654 up-regulated and 512 down-regulated genes were obtained between VST and *VS* at OD_600_ = 0.6. When the OD_600_ increased to 1.2, 1,018 DEGs with 565 up-regulated and 453 down-regulated genes were detected. At the condition of OD_600_ = 2.0, there were 638 up-regulated and 472 down-regulated DEGs (Supplementary Figure S1A; Supplementary Tables S1–S4). KEGG analysis revealed a total of 66 pathways were significantly enriched in the three stages (Supplementary Figure S1B; Supplementary Tables S5–S7), the common pathways including 7 pathways for ribosome synthesis, purine metabolism, biofilm formation, homologous recombination, histidine metabolism, tyrosine metabolism, and amino acid synthesis (Supplementary Figure S1B). Interactive Pathways Explorer (iPath3.0) analysis revealed that the VST DEGs were enriched in most metabolic pathways, in which carbohydrate metabolism and nucleotide metabolism were the most significant ones (Supplementary Figure S1C). Notably, the purine metabolism genes of *purM, purB, purH,* and *gmk* were significantly down-regulated (green numbers, *p* < 0.05) in tetracycline-induced persister cells (Supplementary Figures S1D,E). The expression of these genes were further validated by qRT-PCR, and found that they showed similar depressed expression profiles to transcriptome analysis between tetracycline-induced persister cells and active cells (Supplementary Figure S2).

**Figure 1:**
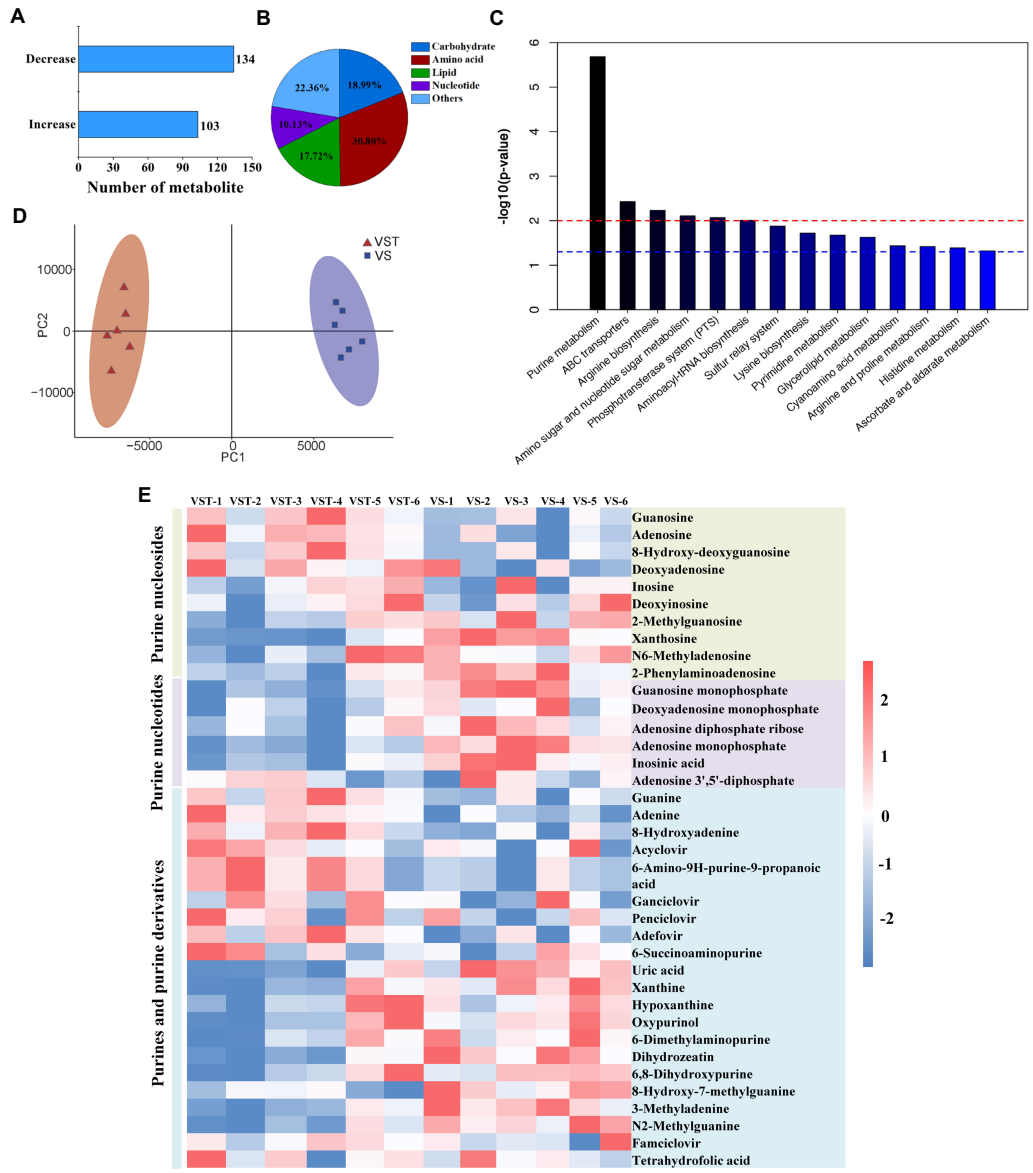
Metabonome analysis of tetracycline-induced persister cells (VST) and active cells (*VS*). The number of the differential metabolites were analyzed by LC–MS **(A)**, and the abundance of metabolic products were clustered into five categories **(B)**. PCA score plot analysis revealed that there was a distinction between VST group of red and *VS* group of purple **(C)**. The pathway of differential metabolite enrichment was indicated by KEGG analysis (*p* < 0.05) **(D)**. **(E)** Heat map for relative abundances of purine metabolites in VST compared with VS. Red and blue indicated increased and decreased metabolite levels relative to the median metabolite level, respectively (see the color scale).

Building on the transcriptome results, liquid chromatography-mass spectroscopy-based metabolome analysis was conducted for VST and *VS* at OD_600_ = 1.2. A total of 237 metabolites were identified as differential produts (Figure 1A), in which 19.0, 30.8, 17.7, and 10.1% abundance were categorized as carbohydrates, amino acids, lipids, and nucleotides, respectively (Figures 1B). The two groups of VST and *VS* were individually clustered together by PCA analysis, demonstrating that tetracycline-induced persister cells and active cells had a distinct metabolic signature (Figure 1C). To explore the metabolic pathways that distinguish VST and *VS*, KEGG was performed to analyze the metabolites of differential abundance. Fourteen metabolic pathways were enriched as shown in Figure 1D and Supplementary Table S8. The six common pathways were purine metabolism (*p* = 2.061 × 10^−6^), ABC transporters (*p* = 0.0037), arginine biosynthesis (*p* = 0.0058), amino sugar and nucleotide sugar metabolism (*p* = 0.0078), phosphotransferase system (PTS) (*p* = 0.0085), and aminoacyl-tRNA biosynthesis (*p* = 0.0098) (Figure 1D). The purine metabolism pathway was the most enriched. By comparing the metabolites of purine metabolism pathway of VST and *VS*, 40 metabolites had significant differential abundance, the identified metabolites were categorized as purine metabolism (25%), purine nucleosides (20%), purines and purine derivatives (55%) (Figure 1E). The purine metabolites of tetracycline-induced persister cells decreased by 57.5% compared with active cells, there are 22 metabolites, including 2-Methyladenosine, Xanthosine, N6-Methyladenosine, 2-Phenylaminoadenosine, Guansine monopjpsphate, and Dexyadenosine monophosphate, were decreased (Figure 1E and Supplementary Table S9). Meanwhile, the intracellular ATP level of tetracycline-induced persister cells was significantly lower than that of active cells (Supplementary Figure S3).

### Reducing purine metabolism promotes AJ01 persistence and inhibits the synthesis of ATP

**Figure 2:**
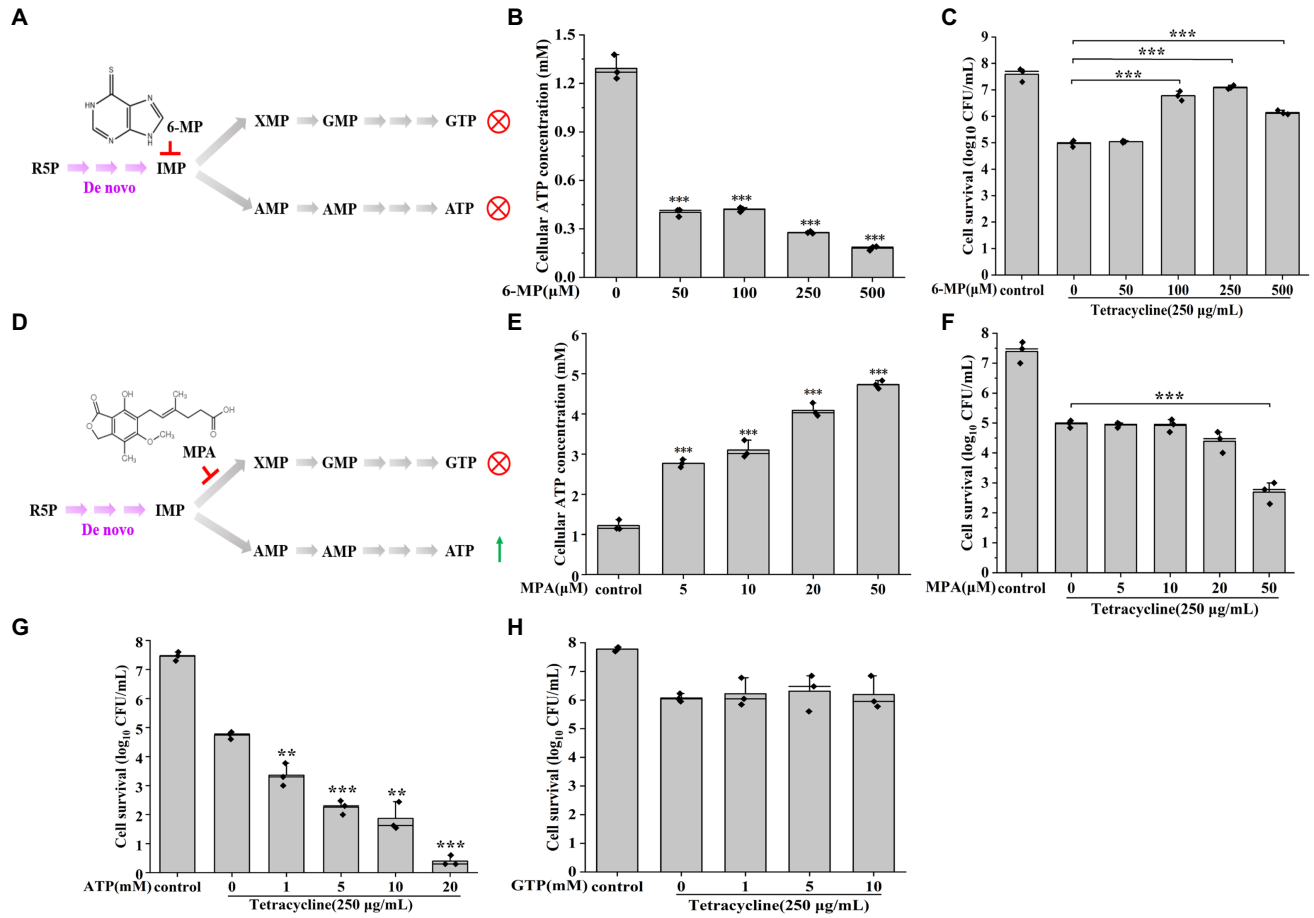
Reducing purine metabolism promotes AJ01 persistence. **(A)** Schematic diagram of 6-MP inhibiting ATP and GTP synthesis. R5P: Ribose 5-phosphate; IMP: inosine monophosphate; GTP: Guanosine-5′-triphosphate; ATP: Adenosine triphosphate; IMPDH: Inosine-5′-monophosphate dehydrogenase; HGPRT: hypoxanthine-guanine phosphoribosyltransferase; 6-MP: 6-Mercaptopurine. **(B)** Intracellular ATP concentration of cells treated with different concentrations of 6-MP. **(C)** Persister cells formation frequency after different concentrations of 6-MP treatment. **(D)** Schematic diagram of MPA inhibiting GTP synthesis. MPA: mycophenolic acid. **(E)** Intracellular ATP concentration of cells treated with different concentrations of MPA. **(F)** Persister cells formation frequency after different concentrations of MPA treatment. **(G)** Persister cells formation frequency after increasing concentrations of exogenous ATP treatment. **(H)** Persister cells formation frequency after increasing concentrations of exogenous GTP treatment. The bars indicated the mean of at least three independent experiments; standard deviation indicated STDEV. (*p value < 0.05; **p value < 0.01; ***p value < 0.001).

To further verify that the down-regulated purine metabolism was associated with persister cells formation, the change of persister cell formation was investigated using different purine metabolism inhibitors (Figure 2). We found that ATP levels were significantly reduced after 6-mercaptopurine (6-MP, inhibits ATP production) treatment (Figures 2A,B), and that a higher ratio of persister cells was also detected from 100 μM 6-MP to 500 μM 6-MP treatments (Figure 2C). On the contrary, inhibiting GTP synthesis *via* MPA treatment significantly increased intracellular ATP levels (Figures 2D,E), and reduced persister cell formation in a dose-dependent manner (Figure 2F). Similarly, the number of persister cells were also decreased after supplying exogenous ATP (Figure 2G), but there were no significant change in persister cell number with exogenous GTP (Figure 2H). All these results support that the depressed purine metabolism, i.e., the ATP synthesis pathway, promote the formation of persister cell in AJ01.

### Reducing purine metabolism accompanies protein aggresome formation

**Figure 3:**
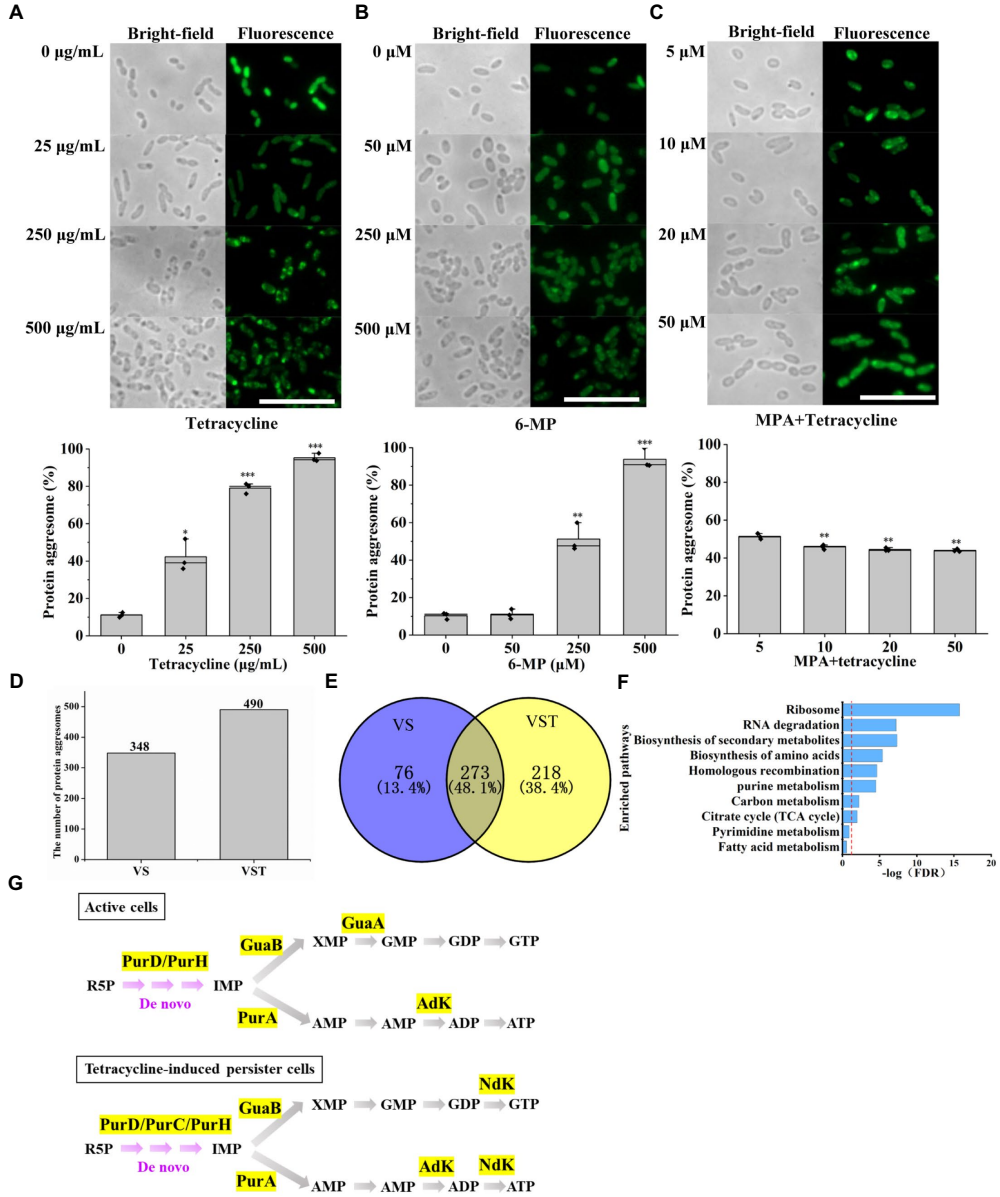
Decreased purine metabolism accompanies protein aggresome formation. Bright-field and fluorescence images showing that protein aggresomes are induced by different concentrations of tetracycline **(A)** or 6-MP **(B)**. And percentage of cells with protein aggresome after different concentrations of tetracycline treatment or 6-MP. On contrast, MPA exposure **(C)** significantly depressed the protein aggresome intensity. Protein aggresome from and tetracycline-induced persister cell and active cells were characterized by mass-spectrometric analysis **(D)**. **(E)** Venn diagram showing the insoluble protein overlap in active cells and tetracycline-induced persister cells. Further KEGG analysis revealed that 273 common proteins were involved in several important biological processes, in which purine metabolism related proteins were showed in **(F)**. **(G)** Purine metabolism-related proteins were found in protein aggresomes of tetracycline-induced persister cells and active cells. The scale bar of the figure was 10 μM. The bars indicated the mean of percentage of cells with protein aggresome in cell of different treatment at least three independent experiments; standard deviation indicated STDEV. (*p value < 0.05; **p value < 0.01; ***p value < 0.001). The microscopy settings were the same across all the images.

In our previous work, we found that a novel phenotypic feature-dark foci was detected in tetracycline-induced persister cells but was not in active cells (Li et al., 2021). To further confirm reducing purine metabolism promoted AJ01 persistence that was related to the protein aggresome formation, we counted the proportion of bacteria that formed protein aggresomes by normalized to cell numbers by microscopy observation. Accordingly, the fraction of cells with protein aggresomes, as assessed by the presence of dark foci or fluorescent foci. The percentages of cells with dark foci after treatment with different concentrations of tetracycline were 11.2, 42.3, 79.1, and 95.3%, respectively (Figure 3A). Within the decreased ATP levels by 6-MP treatment group, the proportion of cells containing protein aggresomes, increased with 6-MP concentration, such as 11.5, 13.9, 46.2, and 90.1%, respectively (Figure 3B). In contrast, increased ATP levels by supplying exogenous MPA significantly reduced the percentages of cells with protein aggresome (Figure 3C). Similarly, the direct addition of 20 mM exogenous ATP also reduced the proportion of cells containing protein aggresomes (Supplementary Figure S4).

Subsequently, we extracted the insoluble protein aggresomes, a total of 490 and 348 insoluble proteins were obtained from tetracycline-induced persister cells and active cells, respectively (Figure 3D and Supplementary Table S10). Among them, analysis of common proteins using Venn, tetracycline-induced persister cells and active cells identified 273 proteins (Figure 3E). KEGG pathway analysis revealed that most proteins aggregated are ribosome proteins and proteins associated with metabolic pathways (Figure 3F). Strikingly, six proteins of PurD, PurH, PurA, GuaB, GuaA, and AdK associated with the purine metabolism were detected in active cells (Figure 3G), and seven proteins of PurD, PurC, PurH, PurA, GuaB, Ndk, and Adk were also detected in tetracycline-induced persister cells (Figure 3G), and the abundance of these proteins were shown in Supplementary Table S11. Protein NdK and PurC were aggregated only in tetracycline-induced persister cells, and its protein abundance was 0.73 and 0.44, respectively (Supplementary Table S11). The main function of NdK and PurC were to maintain the balance of ATP and NTP concentration in cells through phosphate group transfer reaction and to participate in the UTP and GTP biosynthesis process at the same time (Francois-Moutal et al., 2014). These results confirm that tetracycline-induced persister cells with protein aggresome formation that is associated with purine metabolism inhibition and ATP levels reduction.

### Inhibition of purine metabolism is associated with decreased intracellular tetracycline via activation of membrane potential-dependent efflux

**Figure 4:**
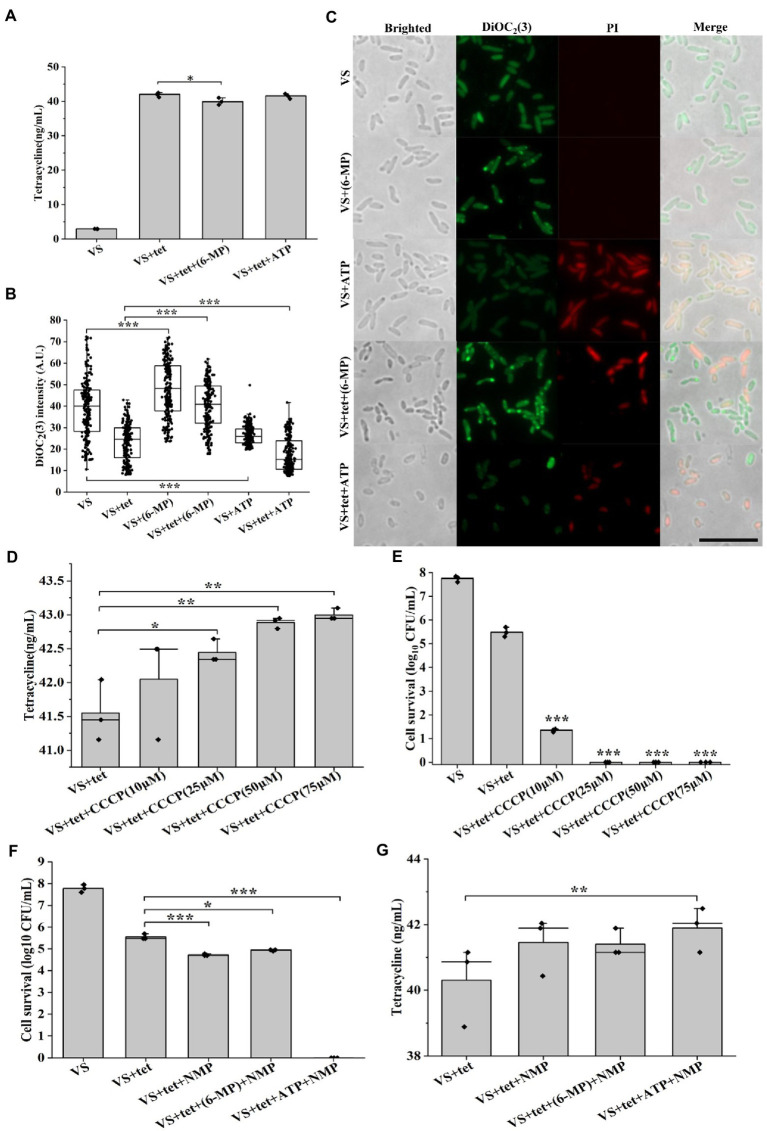
Inhibition of purine metabolism reduces intracellular tetracycline accumulation. Intracellular tetracycline concentration **(A)** and fluorescence intensity of membrane potential **(B, C)** of cells were assayed in the presence of 6-MP/ATP (*n* = 200 for each group). Scale bar, 10 μM. Intracellular tetracycline concentrations of cells (D) and persister cells formation frequency (E) were further assayed in treated with different concentrations of membrane potential inhibitor CCCP. After inhibition of efflux pump by NMP, persister cells formation frequency **(F)** and intracellular tetracycline concentration **(G)** were investigated in 6-MP/ATP exposure conditions. The bars indicated the mean of at least three independent experiments; standard deviation indicated STDEV. (*p value < 0.05; **p value < 0.01; ***p value < 0.001). The microscopy settings were the same across all the images.

**Figure 5:**
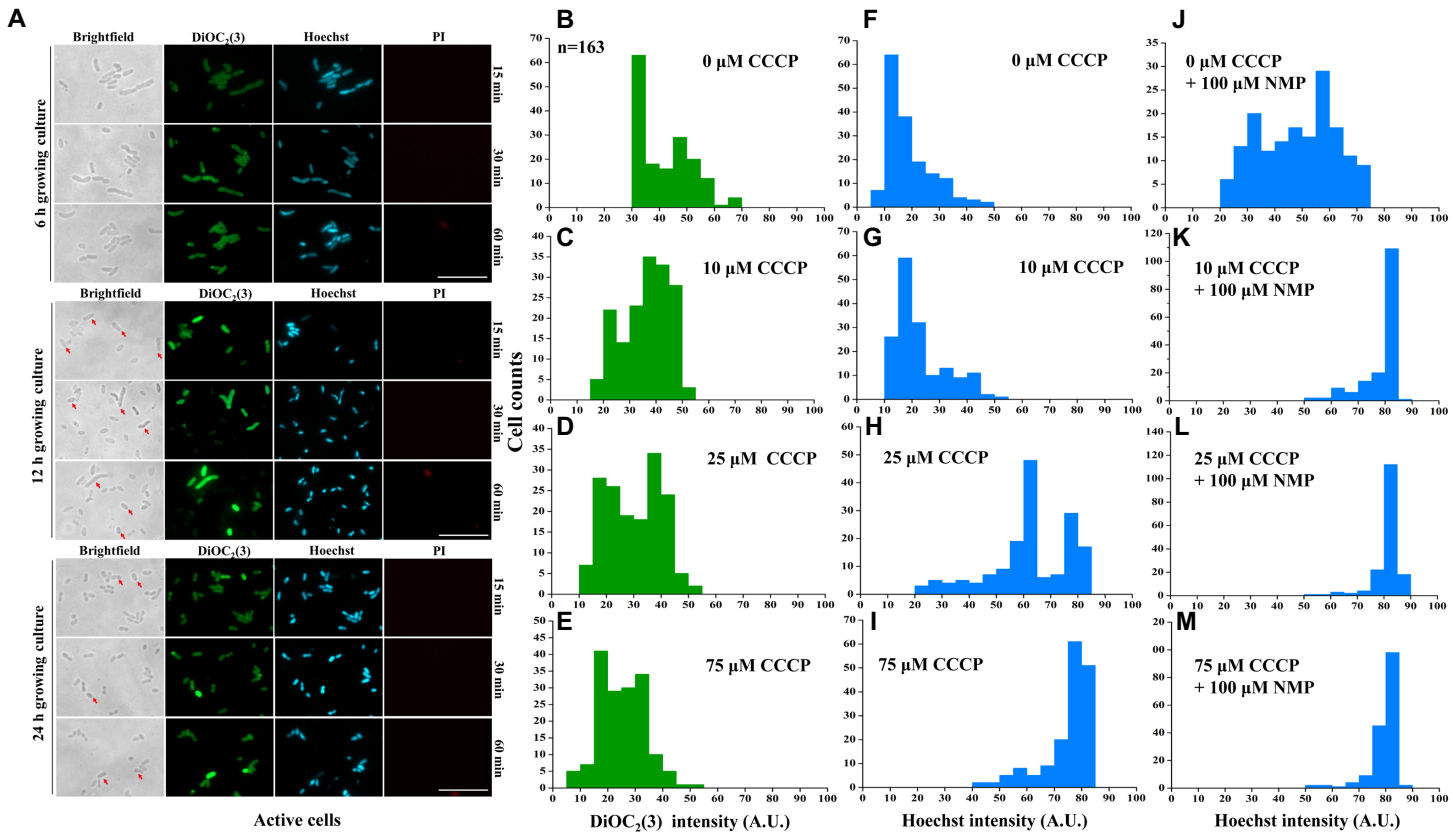
Inhibition of purine metabolism is associated with intracellular tetracycline accumulation *via* activation of membrane potential-dependent efflux. The negative correlation was detected between DiOC_2_(3) and Hoechst 33342 fluorescence intensities in active cells **(A)**. The distribution of DiOC_2_(3) **(B–E)** and Hoechst 33342 fluorescence intensity **(F–I)** was further assayed with different concentrations of CCCP treatments. Similarly, Hoechst 33342 fluorescence intensity was also anlyzed after efflux pump inhibitor NMP treatment **(J–M)**. More than 167 cells were analyzed for each condition. The scale bar of the figure was 10 μM. The microscopy settings were the same across all the images.

Reduction in the intracellular antibiotic accumulation is another factor to promote the formation of persister cells (Roy et al., 2021). To confirm the connection between purine metabolism and intracellular tetracycline accumulation, we investigated whether exogenous 6-MP addition decreased the intracellular concentration of tetracycline. Cells treated with 6-MP showed a significant, though no dramatic, decreased in tetracycline that was consistent with our hypothesis (Figure 4A, *p* = 0.045). Firstly, DiOC_2_(3) was used to measure transmembrane potential of bacteria. DiOC_2_(3) exhibits green fluorescence in all bacterial cells, as the dye molecules self-associate at the higher cytosolic concentrations caused by larger membrane potentials, the green fluorescence is brighter than other low membrane potential after the red fluorescence disappears (Jiang et al., 2020). Consistently, higher membrane potential was found upon 6-MP addition as shown by higher DiOC_2_(3) fluorescence intensity (Figures 4B,C), low membrane potential was found upon ATP addition as shown by low DiOC_2_(3) fluorescence dim (Figures 4B,C), membrane protential was increased though marginally by 2.1%. The change showed a trend in 6-MP treated cells; lower intracellular tetracycline concentration and increased membrane potential (Supplementary Figure S5). Meanwhile, the increase of bacteria stained by PI after the addition of ATP indicates that the cells died (Figure 4C). In contrast, CCCP, the inhibitor of membrane potential treatment increased the concentration of tetracycline (Figures 4D, 5B–D), and also decreased the percentage of persister cells (Figure 4E). Together, these results support that the inhibition of purine metabolism is associated with intracellular tetracycline accumulation, which is achieved by increasing membrane potential.

To further address the potential mechanism of membrane potential-mediated intracellular tetracycline concentrations, then we examined how membrane potential affects substrate transport by using a fluorescent dye, Hoechst 33342 (Le et al., 2021). First, dead cells were excluded by PI staining. For active cells, we found that DiOC_2_(3)-bright cells were Hoechst 33342-dim, whereas DiOC_2_(3)-dim cells were Hoechst 33342-bright (Figure 5A). This observation suggested that the coexistence of cells with two distinct Hoechst 33342 intensities was caused by the heterogeneous on membrane potential, suggesting that membrane potential might regulate the efflux of intracellular Hoechst 33342. In contrast, membrane potential inhibitor CCCP treatment decreased DiOC_2_(3) fluorescence intensity in a dose-dependent manner (Figures 5B–E), and also increased Hoechst 33342 fluorescence intensity (Figures 5F–I). Inhibition of efflux pumps by NMP could increase accumulation of Hoechst 33342 fluorescence intensity (Figures 5J–M). These results suggest that the inverse correlation of fluorescence intensity observed between DiOC_2_(3) and Hoechst 33342, thus, the efflux of Hoechst 33342 is driven by membrane potential. After tetracycline treatment, persister cells with bright DiOC_2_(3) fluorescence intensity of also had dim Hoechst 33342 fluorescence intensity (Supplementary Figure S6). NMP could increase accumulation of Hoechst 33342 fluorescence intensity in 6-MP treatment group, which was consistent with NMP treatment group (Supplementary Figure S6D). Similarly, the concentration of intracellular tetracycline after NMP treatment was also increased by 6-MP treatment (Figures 4F,G). Together, these results reveal that membrane potential mediates tetracycline accumulation by effecting efflux.

### Membrane potential-mediated efflux is depending on dissipating transmembrane proton pH gradient

**Figure 6:**
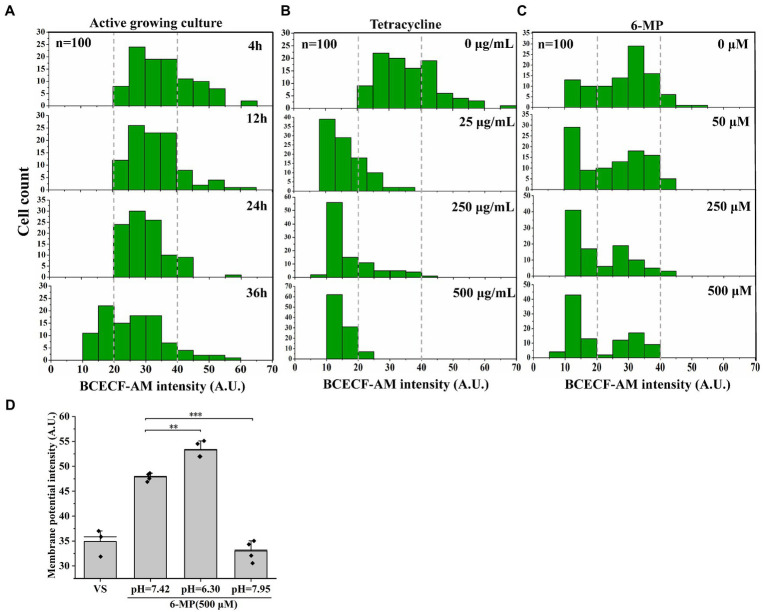
Membrane potential-mediated efflux pump depending on dissipating transmembrane proton pH gradient. BCECF-AM fluorescence intensity was assayed in active cell **(A)**, different concentrations of tetracycline treatment **(B)** (*n* = 100 for each group) or 6-MP treatment **(C)** and the signal value were analyzed by ImageJ. After that, the membrane potential was determined after pH adjustment **(D)** (*n* = 50 for each group). The bars indicated the mean of at least three independent experiments; standard deviation indicated STDEV. (*p value < 0.05; **p value < 0.01; ***p value < 0.001).

The membrane potential and transmembrane proton pH gradient are related (Song et al., 2021). A pH shift in the intracellular microenvironment leads to a decrease in the transmembrane proton pH gradient (Lewis, 2007). We assayed the intracellular pH by using the pH indicator BCECF-AM and found the BCECF-AM fluorescence signal significantly decreased with an increase in tetracycline or 6-MP treatments (Figures 6A–C, and Supplementary Table S12). Similarly, 6-MP treatment also led to a significant increase in DiOC_2_(3) fluorescence in the presence or absence of tetracycline, indicating that the membrane potential was increased (Figure 5B). To further validate the change of membrane potential depending on transmembrane proton pH gradient, the pH of culture was adjusted by adding 40 mM potassium benzoate and 40 mM methylamine hydrochloride (Supplementary Figure S7A). When the pH of the growth medium had been adjusted to be acidic (pH = 6.30), 11% increase of DiOC_2_(3) fluorescence intensity of membrane potential was detected in 6-MP treated bacteria (Figure 6D). In contrast, when the pH was adjusted to alkaline (pH = 7.95), membrane potential was decreased by 38% (Figure 6D). These results support that the inhibition of purine metabolism increases membrane potential depending on dissipation of transmembrane proton pH gradient.

## Discussion

**Figure 7:**
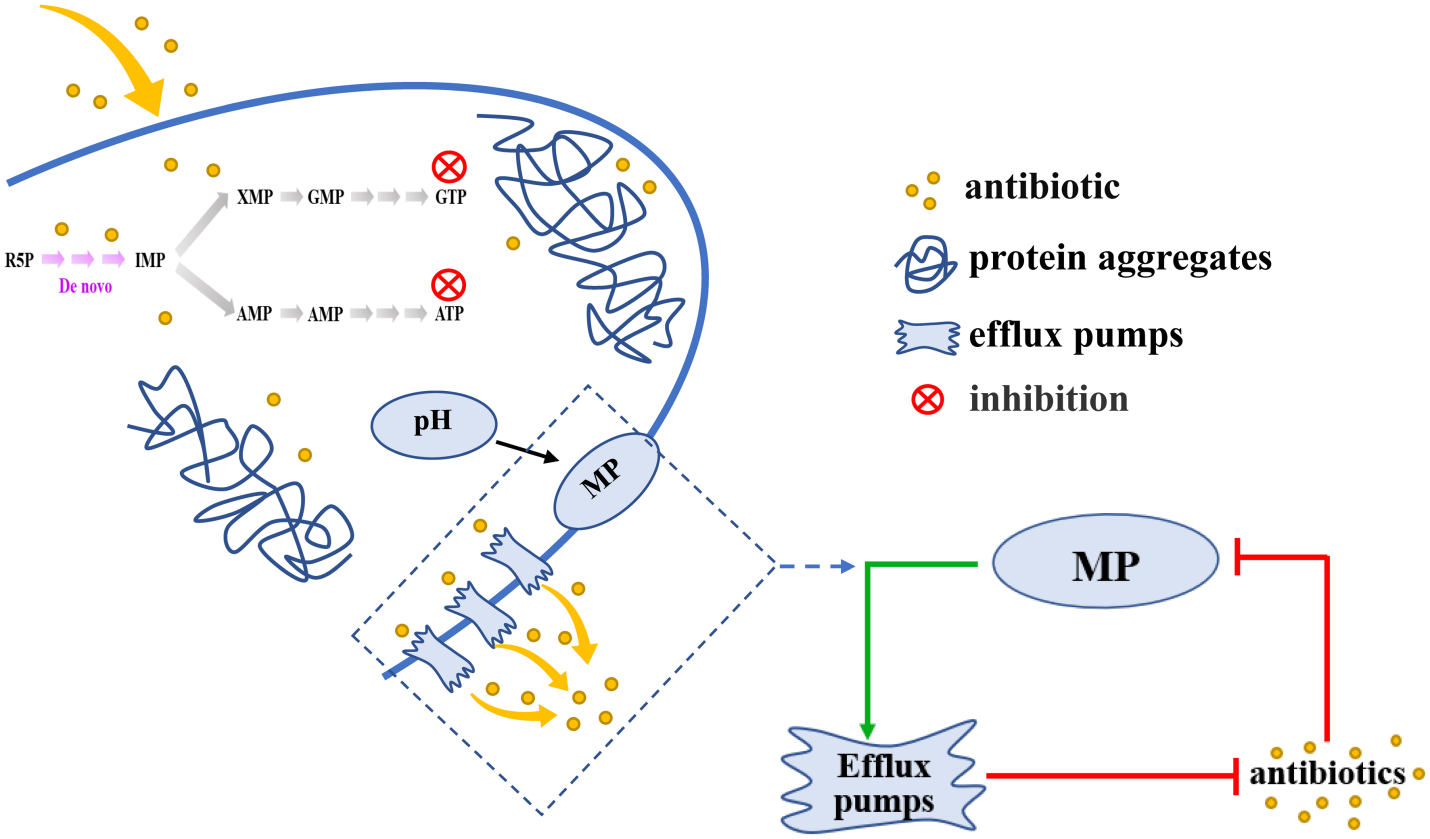
A schematic model for the inhibition of purine metabolism is associated with formation of *V. splendidus* persister cells. Inhibition of Purine metabolism was associated with AJ01 persister cells formation, accompanied by low intracellular ATP levels and the formation of protein aggresome. Meanwhile, the decrease in purine metabolism also affected membrane potential and efflux, reducing intracellular accumulation of tetracycline. The decreased pH dissipated membrane potential (MP) to activate efflux pumps transport antibiotics out of cells. The green arrows mean promotion; red arrows mean inhibition.

Persister cells exhibit an extraordinary tolerance to antibiotics that is dependent on their metabolic state. Although an understanding of persister cell metabolism promises to be a rich source of anti-persistence strategies (Peng et al., 2015), little is known about the mechanisms and phenotypic variation of hypometabolism-induced persister cells. Here, we reported a novel view of inhibition of purine metabolism regulated the formation of AJ01 persister cells. We found that purine metabolism was inhibited in AJ01 persister cells, accompanied by a decreased in intracellular ATP levels, an increased in protein aggresomes formation, and a decreased in the accumulation of intracellular antibiotic concentrations (Figure 7). Specifically, proteins from purine metabolism were identified in aggresomes. Moreover, the intracellular pH was decreased, which increased the membrane potential and led to more efflux of the intracellular antibiotics. Overall, this ensured the non-growing persister cells survive due to lower intracellular levels of antibiotics, meanwhile, the increased efflux by persisters was important for recovery from the persistent state, as the cells resume growth and the antibiotics efflux thereby evading antibiotic killing (Figure 7).

Purines are the most abundant metabolic substrates for all organisms, providing essential components for DNA and RNA synthesis and the necessary energy for cell survival and proliferation (Virgilio and Adinolfi, 2017; Yin et al., 2018). In addition, the inhibition of purine metabolism has been linked to antibiotic resistance in *E. coli*, *Bacillus subtilis*, *Aeromonas veronii* (Sah et al., 2015; Stepanek et al., 2016; Wang et al., 2020). Bacteria are able to take up ATP and GTP *via* general porins (Chaudry et al., 1985; Rudel et al., 1996; Watanabe et al., 2011). The uptaken ATP and GTP may have been converted to adenine and hypoxanthine, which in turn affect ATP levels through purine metabolic pathways (Burton, 1977). And there is feedback regulation between GTP and ATP to regulate ATP levels (Kriel et al., 2012). Wang et al. (2020) showed that increasing ATP levels through purine metabolism in *A. veronii* reduced trimethoprim resistance, which is consistent with our results (Figure 2G); hence inhibiting purine metabolism and ATP production increases the tetracycline resistance of AJ01 persister cells. ATP has also previously been suggested to impact survival to antibiotics, for the decreased ATP would decrease the activity of ATP-dependent antibiotic targets such as gyrase, topoisomerase and RNA polymerase leading to antibiotic resistance (Kwan et al., 2013; Conlon et al., 2016). Reduced ATP would reduce the growth rate, making antibiotics less effective since the cells are not rapidly growing. A report found that lowering intracellular ATP in a growing population to stationary levels with arsenate treatment strongly increased the level of persister cells tolerant to fluoroquinolones (Shan et al., 2017).

Protein aggresomes have been associated with *E. coli* persister cells (Pu et al., 2018; Bollen et al., 2021). ATP acts as a biological hydrotrope to maintain protein solubility and prevent macromolecular aggregation (Patel et al., 2017), by facilitating protein folding and disaggregation, because protein chaperones and proteases (involved in proper folding and degradation of misfolded proteins) require ATP (Patel et al., 2017). Similarly, in our case, we found that the reduction in purine metabolism to ATP caused by 6-MP was associated with persister cell formation and protein aggresome formation (Figure 3B). Consistently, *E. coli* persister cells in nutrient-depleted conditions (low ATP levels) have more protein aggresome than nondormant cells (Pu et al., 2018). Moreover, the amount of aggregated proteins appears to be correlated to dormancy depth at the single-cell level, with shallowly dormant persister cells having less aggregates (Bollen et al., 2021). Consistent with this observation, when dormancy depth was increased by culturing cells for longer at stationary phase, the fraction of insoluble protein in the cell also increased in *E. coli* (Pu et al., 2018). Protein aggregation leads to the loss of function of multiple proteins and serves to inactivate metabolism, which causes bacterial persistence to external stresses (Bollen et al., 2021). Ribosomes were found in tetracycline-induced protein aggresomes, which serve as the targets for antibiotic binding sites (Arenz et al., 2015). In our protein aggresome analysis, the ribosome-and the purine-metabolism-related proteins were also identified (Figure 3F and Supplementary Table S10), supporting that protein aggresome might increase antibiotic resistance by inactivating tetracycline targets.

Bacteria can maintain persistence by lowering intracellular antibiotic concentrations *via* efflux (Zou et al., 2022). Similarly, we found that the persister cells with 6-MP treatment had low intracellular antibiotic concentrations compared to the active cells (Figures 4A, *p* = 0.045). Meanwhile, higher membrane potential was found upon 6-MP addition and low membrane potential was found upon ATP addition (Figures 4B,C), implied that inhibition of purine metabolism by 6-MP could regulate membrane potential. Inhibition of efflux by NMP treatment also increased the accumulation of intracellular tetracycline and reduced AJ01 persistence (Figures 4F,G). The function of bacterial efflux pumps is influenced by the membrane potential (Krulwich et al., 2011). The reduced membrane potential of *Klebsiella pneumoniae* inhibited efflux, weakened resistance to tigecycline and promoted bacterial death (Sun et al., 2021). The membrane potential for efflux to transport 3,3,4,5-tetrachlorosalicylanilide from the cells and protects *E. coli* from the harmful effects of antibiotics (Le et al., 2021). In our case, inhibiting the membrane potential *via* CCCP, increases tetracycline and reduces the number of persister cells (Figures 4D,E). Also, the membrane potential and transmembrane proton pH gradient have an important compensation mechanism in the bacterial membrane (Martins et al., 2009). We found that inhibition of purine metabolism was associated with a higher membrane potential as a result of dissipation of the transmembrane proton pH gradient, thus the concentration of tetracycline was reduced (Figure 6), which was consistent with results from drug-resistance bacteria of *E. coli* and *S. aureus* (Hards et al., 2018; Lu et al., 2021). In addition, pH can be altered by antibiotics by regulating H^+^/K^+^ ions (Hards et al., 2018; Lu et al., 2021). Similarly, the pH gradually decreased with the increase of the concentration of tetracycline or 6-MP in our study (Figures 6C,D). How tetracycline and 6-MP affect the change of intracellular pH should be explored in future research. Interestingly, using a microscope for single-cell observations, the persister cells with protein aggregation had lower pH value than the cells without protein aggregation (Supplementary Figures S7B,C). Munder et al. (2016) reported that nutrient scarcity caused cellular ATP depletion and a drop in cytosolic pH, which in turn promoted macromolecule granule formation and cytoplasmic solidification, therefore enhancing cell persistence. In order to tested whether a drop in intracellular pH is causal to promote protein aggresome formation. The 8.22, 6.87, and 6.31 is adjustment of the pH of the 2216E fluid medium. The results showed that there was no significant difference in the percentages of cells with protein aggresome with different intracellular pH values (Supplementary Figure S7D). We found that an acidic cytoplasm was not the reason that causes protein aggresome formation in bacterial cells. Instead, our results are similar to those of Pu et al. (2018). Therefore, the intracellular pH has an important impact on the physiology and phenotype of cells. Whether intracellular pH is related to protein aggresome formation should be investigated further.

The formation mechanism of persister cell is complex. When cells face the pressure of antibiotics, they will systematically inhibit multiple metabolic activities, making cells gradually develop toward persistent (Amato et al., 2013; Amato and Brynildsen, 2015). In this study, although we have demonstrated that inhibition of bacterial purine metabolism have a important role in promoting cell persistence and accompany by a decrease in intracellular ATP levels and an increase in protein aggresome formation. These phenotypic and physiological characteristics were favorable conditions to promote the formation of persisters in previous studies (Conlon et al., 2016; Pu et al., 2016; Shan et al., 2017; Pu et al., 2018). But the regulatory relationship among protein aggresome formation and perisister cell formation needed to be further validated. This is consistent with the question of whether ATP depletion and protein aggresome formation are interdependent, as reported by Pu et al. (2018). More precisely, the inhibition of purine metabolism is closely related to the formation of persisters in this study. Meanwhile, intracellular accumulation of tetracycline also appears to have some relevance to purine metabolism. Inhibition of purine metabolism results in lower intracellular antibiotic concentrations, suggesting that purine metabolism play a potential role in the regulation of intracellular antibiotic efflux. It remains to be explored whether the inhibition of bacterial purine metabolism has more advantages than other possible mechanisms for persister cells formation.

## Data availability statement

The original contributions presented in the study are included in the article/Supplementary material, further inquiries can be directed to the corresponding author. The RNA-seq data presented in the study are deposited in the Gene Expression Omnibus (GEO) repository, accession number GSE217630. The metabolite data presented in the study are deposited in the MetaboLights repository, accession number MTBLS6405.

## Author contributions

YL: investigation, methodology, formal analysis, data curation, validation, visualization, writing-original draft, and writing – review and editing. TW: methodology, writing – review and editing. WZ: methodology, formal analysis, supervision, validation, and writing – review and editing. CL: conceptualization, data curation, investigation, methodology, formal analysis, resources, supervision, validation, writing – review and editing, project administration, and funding acquisition. All authors contributed to the article and approved the submitted version.

## Funding

This work was supported by Natural Science Foundation of Zhejiang Province (LZ19C190001), National Natural Science Foundation of China (32073003), and the K.C. Wong Magna Fund in Ningbo University.

## Conflict of interest

The authors declare that the research was conducted in the absence of any commercial or financial relationships that could be construed as a potential conflict of interest.

## Publisher’s note

All claims expressed in this article are solely those of the authors and do not necessarily represent those of their affiliated organizations, or those of the publisher, the editors and the reviewers. Any product that may be evaluated in this article, or claim that may be made by its manufacturer, is not guaranteed or endorsed by the publisher.

## Footnotes

^1^ https://pathways.embl.de/

^2^ https://bioinfogp.cnb.csic.es/tools/venny/index.html

^3^ http://kobas.cbi.pku.edu.cn/index.php

## References

[ref1] Alekshun M. N. Levy S. B. (2007). Molecular mechanisms of antibacterial multidrug resistance. Cells 128, 1037–1050. doi: 10.1016/j.cell.2007.03.004 17382878

[ref2] Amato S. M. Brynildsen M. P. (2015). Persister heterogeneity arising from a single metabolic stress. Curr. Biol. 25, 2090–2098. doi: 10.1016/j.cub.2015.06.034, PMID: 26255847

[ref3] Amato S. M. Orman M. A. Brynildsen M. P. (2013). Metabolic control of persister formation in *Escherichia coli*. Mol. Cell 50, 475–487. doi: 10.1016/j.molcel.2013.04.002 23665232

[ref4] Anderson G. G. Palermo J. J. Schilling J. D. Roth R. Heuser J. Hultgren S. J. (2003). Intracellular bacterial biofilm-like pods in urinary tract infections. Science 301, 105–107. doi: 10.1126/science.1084550, PMID: 12843396

[ref5] Arenz S. Nguyen F. Beckmann R. Wilson D. N. (2015). Cryo-EM structure of the tetracycline resistance protein TetM in complex with a translating ribosome at 3.9-Å resolution. Proc. Natl. Acad. Sci. U. S. A. 112, 5401–5406. doi: 10.1073/pnas.1501775112, PMID: 25870267 PMC4418892

[ref6] Blundell R. D. Williams S. J. Arras S. D. Chitty J. L. Blake K. L. Ericsson D. J.. (2016). Disruption of de novo adenosine triphosphate (ATP) biosynthesis abolishes virulence in *Cryptococcus neoformans*. ACS Infect. Dis. 2, 651–663. doi: 10.1021/acsinfecdis.6b00121, PMID: 27759389

[ref7] Bollen C. Dewachter L. Michiels J. (2021). Protein aggregation as a bacterial strategy to survive antibiotic treatment. Front. Mol. Biosci. 8:669664. doi: 10.3389/fmolb.2021.669664, PMID: 33937340 PMC8085434

[ref8] Burton K. (1977). Transport of adenine, hypoxanthine and uracil into *Escherichia coli*. Biochem. J. 168, 195–204. doi: 10.1042/bj1680195, PMID: 413544 PMC1183752

[ref9] Byrd B. A. Zenick B. Rocha-Granados M. C. Englander H. E. Hare P. J. LaGree T. J.. (2021). The AcrAB-TolC efflux pump impacts persistence and resistance development in stationary-phase *Escherichia coli* following delafloxacin treatment. Antimicrob. Agents Chemother. 65:e0028121. doi: 10.1128/AAC.00281-21, PMID: 34097492 PMC8284433

[ref10] Camp J. Schuster S. Vavra M. Schweigger T. Rossen J. W. A. Reuter S.. (2021). Limited multidrug resistance efflux pump overexpression among multidrug-resistant *Escherichia coli* strains of ST131. Antimicrob. Agents Chemother. 65, e01735–e01720. doi: 10.1128/AAC.01735-20, PMID: 33468485 PMC8097430

[ref11] Chaudry I. H. Clemens M. G. Baue A. E. (1985). “Uptake of ATP by tissues” in Purines. Satellite Symposia of the IUPHAR 9th International Congress of Pharmacology. ed. Stone T. W. (London: Palgrave Macmillan)

[ref12] Cheng H. Y. Soo V. W. Islam S. McAnulty M. J. Benedik M. J. Wood T. K. (2014). Toxin GhoT of the GhoT/GhoS toxin/antitoxin system damages the cell membrane to reduce adenosine triphosphate and to reduce growth under stress. Environ. Microbiol. 16, 1741–1754. doi: 10.1111/1462-2920.12373, PMID: 24373067

[ref13] Conlon B. P. Rowe S. E. Gandt A. B. Nuxoll A. S. Donegan N. P. Zalis E. A.. (2016). Persister formation in *Staphylococcus aureus* is associated with ATP depletion. Nat. Microbiol. 1:16051. doi: 10.1038/nmicrobiol.2016.51, PMID: 27572649 PMC4932909

[ref14] Donner J. Reck M. Bunk B. Jarek M. App C. B. Meier-Kolthoff J. P.. (2017). The biofilm inhibitor carolacton enters gram-negative cells: studies using a TolC-deficient strain of *Escherichia coli*. mSphere 2, e00375–e00317. doi: 10.1128/mSphereDirect.00375-17, PMID: 28959742 PMC5615136

[ref15] Dörr T. Lewis K. Vulić M. (2009). SOS response induces persistence to fluoroquinolones in *Escherichia coli*. PLoS Genet. 5:e1000760. doi: 10.1371/journal.pgen.1000760, PMID: 20011100 PMC2780357

[ref16] Francois-Moutal L. Marcillat O. Granjon T. (2014). Structural comparison of highly similar nucleoside-diphosphate kinases: molecular explanation of distinct membrane-binding behavior. Biochimie 105, 110–118. doi: 10.1016/j.biochi.2014.06.025, PMID: 25010650

[ref17] Germain E. Roghanian M. Gerdes K. Maisonneuve E. (2015). Stochastic induction of persister cells by HipA through (p)ppGpp-mediated activation of mRNA endonucleases. Proc. Natl. Acad. Sci. U. S. A. 112, 5171–5176. doi: 10.1073/pnas.1423536112, PMID: 25848049 PMC4413331

[ref18] Gollan B. Grabe G. Michaux C. Helaine S. (2019). Bacterial persisters and infection: past, present, and progressing. Annu. Rev. Microbiol. 73, 359–385. doi: 10.1146/annurev-micro-020518-115650, PMID: 31500532

[ref19] Griffith J. M. Basting P. J. Bischof K. M. Wrona E. P. Kunka K. S. Tancredi A. C.. (2019). Experimental evolution of *Escherichia coli* K-12 in the presence of proton motive force (PMF) uncoupler carbonyl cyanide mchlorophenylhydrazone selects for mutations affecting PMF-driven drug efflux pumps. Appl. Environ. Microbiol. 85, e02792–e02718. doi: 10.1128/AEM.02792-18, PMID: 30578262 PMC6384104

[ref20] Hardie D. G. Ross F. A. Hawley S. A. (2012). AMPK: a nutrient and energy sensor that maintains energy homeostasis. Nat. Rev. Mol. Cell Biol. 13, 251–262. doi: 10.1038/nrm3311, PMID: 22436748 PMC5726489

[ref21] Hards K. McMillan D. G. G. Schurig-Briccio L. A. Gennis R. B. Lill H. Bald D.. (2018). Ionophoric effects of the antitubercular drug bedaquiline. Proc. Natl. Acad. Sci. U. S. A. 115, 7326–7331. doi: 10.1073/pnas.1803723115, PMID: 29941569 PMC6048524

[ref22] Holden D. W. Errington J. (2018). Type II toxin-antitoxin systems and persister cells. MBio 9, e01574–e01518. doi: 10.1128/mBio.01574-18 30254124 PMC6156201

[ref01] Hu P. Li G. Zhao X. Zhao F. Li L. Zhou H. (2018). Transcriptome profiling by RNA-Seq reveals differentially expressed genes related to fruit development and ripening characteristics in strawberries (Fragaria × ananassa). PeerJ. 6:e4976. doi: 10.7717/peerj.4976 29967718 PMC6026456

[ref23] Jiang M. Kuang S. F. Lai S. S. Zhang S. Yang J. Peng B.. (2020). Na-NQR confers aminoglycoside resistance via the regulation of L-alanine metabolism. MBio 11, e02086–e02020. doi: 10.1128/mBio.02086-20 33203750 PMC7683393

[ref02] Jin Y. Hammell M. (2018). Analysis of RNA-Seq data using TEtranscripts. Methods Mol. Biol. 1751, 153–167. doi: 10.1007/978-1-4939-7710-9_11 29508296

[ref24] Kaldalu N. Hauryliuk V. Turnbull K. J. La L. E. M. A. Putrinš M. Tenson T. (2020). In vitro studies of persister cells. Microbiol. Mol. Biol. Rev. 84, e00070–e00020. doi: 10.1128/MMBR.00070-20, PMID: 33177189 PMC7667008

[ref25] Keren I. Minami S. Rubin E. Lewis K. (2011). Characterization and transcriptome analysis of mycobacterium tuberculosis persisters. mBio 2, e00100–e00111. doi: 10.1128/mBio.00100-11 21673191 PMC3119538

[ref26] Khan F. Pham Dung T. N. Tabassum N. Oloketuyi S. F. Kim Y. M. (2020). Treatment strategies targeting persister cell formation in bacterial pathogens. Crit. Rev. Microbiol. 46, 665–688. doi: 10.1080/1040841X.2020.1822278, PMID: 33022189

[ref27] Kim B. H. Gadd G. M. (2019). Membrane Transport-Nutrient Uptake and Protein Excretion, Cambridge University Press, England.

[ref28] Kriel A. Bittner A. N. Kim S. H. Liu K. Tehranchi A. K. Zou W. Y.. (2012). Direct regulation of GTP homeostasis by (p)ppGpp: a critical component of viability and stress resistance. Mol. Cell 48, 231–241. doi: 10.1016/j.molcel.2012.08.009, PMID: 22981860 PMC3483369

[ref29] Krishnan M. Y. Manning E. J. Collins M. T. (2009). Effects of interactions of antibacterial drugs with each other and with 6-mercaptopurine on in vitro growth of *Mycobacterium avium* subspecies paratuberculosis. J. Antimicrob. Chemother. 64, 1018–1023. doi: 10.1093/jac/dkp339, PMID: 19759042

[ref30] Krulwich T. A. Sachs G. Padan E. (2011). Molecular aspects of bacterial pH sensing and homeostasis. Nat. Rev. Microbiol. 9, 330–343. doi: 10.1038/nrmicro2549, PMID: 21464825 PMC3247762

[ref31] Kwan B. W. Valenta J. A. Benedik M. J. Wood T. K. (2013). Arrested protein synthesis increases persister-like cell formation. Antimicrob. Agents Chemother. 57, 1468–1473. doi: 10.1128/AAC.02135-12, PMID: 23295927 PMC3591907

[ref32] LaFleur M. D. Qi Q. Lewis K. (2010). Patients with long-term oral carriage harbor high-persister mutants of *Candida albicans*. Antimicrob. Agents Chemother. 54, 39–44. doi: 10.1128/AAC.00860-09, PMID: 19841146 PMC2798516

[ref33] Langmead B. Salzberg S. L. (2012). Fast gapped-read alignment with Bowtie 2. Nat. Methods 9, 357–359. doi: 10.1038/nmeth.1923, PMID: 22388286 PMC3322381

[ref34] Le D. Krasnopeeva E. Sinjab F. Pilizota T. Kim M. (2021). Active efflux leads to heterogeneous dissipation of proton motive force by protonophores in bacteria. MBio 12:e0067621. doi: 10.1128/mBio.00676-21 34253054 PMC8406135

[ref35] Lewis K. (2007). Persister cells, dormancy and infectious disease. Nat. Rev. Microbiol. 5, 48–56. doi: 10.1038/nrmicro1557 17143318

[ref36] Li L. Su Y. B. Peng B. Peng X. X. Li H. (2020). Metabolic mechanism of colistin resistance and its reverting in *Vibrio alginolyticus*. Environ. Microbiol. 22, 4295–4313. doi: 10.1111/1462-2920.15021, PMID: 32291842

[ref37] Li Y. Wood T. K. Zhang W. Li C. (2021). *Vibrio splendidus* persister cells induced by host coelomic fluids show a similar phenotype to antibiotic-induced counterparts. Environ. Microbiol. 23, 5605–5620. doi: 10.1111/1462-2920.15717, PMID: 34390618

[ref38] Lu C. H. Shiau C. W. Chang Y. C. Kung H. N. Wu J. C. Lim C. H.. (2021). SC5005 dissipates the membrane potential to kill *Staphylococcus aureus* persisters without detectable resistance. J. Antimicrob. Chemother. 76, 2049–2056. doi: 10.1093/jac/dkab114, PMID: 33855344

[ref39] Man Z. Guo J. Zhang Y. Cai Z. (2020). Regulation of intracellular ATP supply and its application in industrial biotechnology. Crit. Rev. Biotechnol. 40, 1151–1162. doi: 10.1080/07388551.2020.1813071, PMID: 32862717

[ref40] Manning B. D. (2013). Adaptation to starvation: translating a matter of life or death. Cancer Cell 23, 713–715. doi: 10.1016/j.ccr.2013.05.012 23763997

[ref41] Martins A. Spengler G. Rodrigues L. Viveiros M. Ramos J. Martins M.. (2009). pH modulation of efflux pump activity of multi-drug resistant *Escherichia coli*: protection during its passage and eventual colonization of the colon. PLoS One 4:e6656. doi: 10.1371/journal.pone.0006656, PMID: 19684858 PMC2722724

[ref42] Moldoveanu A. L. Rycroft J. A. Helaine S. (2021). Impact of bacterial persisters on their host. Curr. Opin. Microbiol. 59, 65–71. doi: 10.1016/j.mib.2020.07.006 32866708

[ref43] Mulcahy L. R. Burns J. L. Lory S. Lewis K. (2010). Emergence of *Pseudomonas aeruginosa* strains producing high levels of persister cells in patients with cystic fibrosis. J. Bacteriol. 192, 6191–6199. doi: 10.1128/JB.01651-09, PMID: 20935098 PMC2981199

[ref44] Munder M. C. Midtvedt D. Franzmann T. Nüske E. Otto O. Herbig M.. (2016). A pH-driven transition of the cytoplasm from a fluid to a solid-like state promotes entry into dormancy. Elife 5:e09347. doi: 10.7554/eLife.09347, PMID: 27003292 PMC4850707

[ref45] Pages J. M. James C. E. Winterhalter M. (2008). The porin and the permeating antibiotic: a selective diffusion barrier in gram-negative bacteria. Nat. Rev. Microbiol. 6, 893–903. doi: 10.1038/nrmicro1994, PMID: 18997824

[ref46] Parry B. R. Surovtsev I. V. Cabeen M. T. O'Hern C. S. Dufresne E. R. Jacobs-Wagner C. (2014). The bacterial cytoplasm has glass-like properties and is fluidized by metabolic activity. Cells 156, 183–194. doi: 10.1016/j.cell.2013.11.028, PMID: 24361104 PMC3956598

[ref47] Patel A. Malinovska L. Saha S. Wang J. Alberti S. Krishnan Y.. (2017). ATP as a biological hydrotrope. Science 356, 753–756. doi: 10.1126/science.aaf6846, PMID: 28522535

[ref48] Paulsen I. T. Brown M. H. Skurray R. A. (1996). Proton-dependent multidrug efflux systems. Microbiol. Rev. 60, 575–608. doi: 10.1128/mr.60.4.575-608.1996, PMID: 8987357 PMC239457

[ref49] Peng B. Su Y. B. Li H. Han Y. Guo C. Tian Y. M.. (2015). Exogenous alanine and/or glucose plus kanamycin kills antibiotic-resistant bacteria. Cell Metab. 21, 249–262. doi: 10.1016/j.cmet.2015.01.008 25651179

[ref50] Polak D. Shany-Kdoshim S. Zaydel L. Feuerstein O. Houri-Haddad Y. (2019). High-resolution novel method for tracking bacteria in a multi-species biofilm. Arch. Microbiol. 201, 259–266. doi: 10.1007/s00203-018-1614-z, PMID: 30610246

[ref51] Pu Y. Ke Y. Bai F. (2017). Active efflux in dormant bacterial cells-new insights into antibiotic persistence. Drug Resist. Updat. 30, 7–14. doi: 10.1016/j.drup.2016.11.002, PMID: 28363336

[ref52] Pu Y. Li Y. Jin X. Tian T. Ma Q. Zhao Z.. (2018). ATP-dependent dynamic protein aggregation regulates bacterial dormancy depth critical for antibiotic tolerance. Mol. Cell 73, 143–156.e4. doi: 10.1016/j.molcel.2018.10.022, PMID: 30472191

[ref53] Pu Y. Zhao Z. Li Y. Zou J. Ma Q. Zhao Y.. (2016). Enhanced efflux activity facilitates drug tolerance in dormant bacterial cells. Mol. Cell 62, 284–294. doi: 10.1016/j.molcel.2016.03.035, PMID: 27105118 PMC4850422

[ref54] Roy S. Bahar A. A. Gu H. Nangia S. Sauer K. Ren D. (2021). Persister control by leveraging dormancy associated reduction of antibiotic efflux. PLoS Pathog. 17:e1010144. doi: 10.1371/journal.ppat.1010144, PMID: 34890435 PMC8716142

[ref55] Rudel T. Schmid A. Benz R. Kolb H. A. Lang F. Meyer T. F. (1996). Modulation of neisseria porin (PorB) by cytosolic ATP/GTP of target cells: parallels between pathogen accommodation and mitochondrial endosymbiosis. Cells 85, 391–402. doi: 10.1016/s0092-8674(00)81117-4, PMID: 8616894

[ref56] Sah S. Aluri S. Rex K. Varshney U. (2015). One-carbon metabolic pathway rewiring in *Escherichia coli* reveals an evolutionary advantage of 10-formyltetrahydrofolate synthetase (Fhs) in survival under hypoxia. J. Bacteriol. 197, 717–726. doi: 10.1128/JB.02365-14, PMID: 25448816 PMC4334196

[ref57] Shan Y. Brown G. A. Rowe S. E. Deisinger J. P. Conlon B. P. Lewis K. (2017). ATP-dependent persister formation in *Escherichia coli*. mBio 8, e02267–e02216. doi: 10.1128/mBio.02267-16 28174313 PMC5296605

[ref58] Song M. Liu Y. Li T. Liu X. Hao Z. Ding S.. (2021). Plant natural flavonoids against multidrug resistant pathogens. Adv. Sci. 8:e2100749. doi: 10.1002/advs.202100749, PMID: 34041861 PMC8336499

[ref59] Stepanek J. J. Schäkermann S. Wenzel M. Prochnow P. Bandow J. E. (2016). Purine biosynthesis is the bottleneck in trimethoprim-treated Bacillus subtilis. Proteomics Clin. Appl. 10, 1036–1048. doi: 10.1002/prca.201600039, PMID: 27329548

[ref60] Sun J. Deng Z. Yan A. (2014). Bacterial multidrug efflux pumps: mechanisms, physiology and pharmacological exploitations. Biochem. Biophys. Res. Commun. 453, 254–267. doi: 10.1016/j.bbrc.2014.05.090, PMID: 24878531

[ref61] Sun L. L. Sun L. Li X. Hu X. X. Wang X. K. Nie T. Y.. (2021). A novel tigecycline adjuvant ML-7 reverses the susceptibility of tigecycline-resistant Klebsiella pneumoniae. Front. Cell. Infect. Microbiol. 11:809542. doi: 10.3389/fcimb.2021.809542, PMID: 35071055 PMC8766836

[ref62] Taber H. W. Mueller J. P. Miller P. F. Arrow A. S. (1987). Bacterial uptake of aminoglycoside antibiotics. Microbiol. Rev. 51, 439–457. doi: 10.1128/mr.51.4.439-457.1987, PMID: 3325794 PMC373126

[ref63] Torrey H. L. Keren I. Via L. E. Lee J. S. Lewis K. (2016). High persister mutants in *Mycobacterium tuberculosis*. PLoS One 11:e0155127. doi: 10.1371/journal.pone.0155127, PMID: 27176494 PMC4866775

[ref64] Van den Bergh B. Fauvart M. Michiels J. (2017). Formation, physiology, ecology, evolution and clinical importance of bacterial persisters. FEMS Microbiol. Rev. 41, 219–251. doi: 10.1093/femsre/fux001, PMID: 28333307

[ref65] Verstraeten N. Knapen W. J. Kint C. I. Liebens V. Van Den Bergh B. Dewachter L.. (2015). Obg and membrane depolarization are part of a microbial bet-hedging strategy tha leads to antibiotic tolerance. Mol. Cell 59, 9–21. doi: 10.1016/j.molcel.2015.05.011, PMID: 26051177

[ref66] Virgilio F. D. Adinolfi E. (2017). Extracellular purines, purinergic receptors and tumor growth. Oncogene 36, 293–303. doi: 10.1038/onc.2016.206, PMID: 27321181 PMC5269532

[ref67] Wang T. El Meouche I. Dunlop M. J. (2017). Bacterial persistence induced by salicylate via reactive. Sci. Rep. 7:43839. doi: 10.1038/srep43839, PMID: 28281556 PMC5345018

[ref68] Wang D. Li H. Khan W. U. Ma X. Tang H. Tang Y.. (2020). SmpB and tmRNA orchestrate purine pathway for the trimethoprim resistance in *Aeromonas veronii*. Front. Cell. Infect. Microbiol. 10:239. doi: 10.3389/fcimb.2020.00239, PMID: 32547961 PMC7270562

[ref69] Watanabe K. Tomioka S. Tanimura K. Oku H. Isoi K. (2011). Uptake of AMP, ADP, and ATP in *Escherichia coli* W. Biosci. Biotechnol. Biochem. 75, 7–12. doi: 10.1271/bbb.100063, PMID: 21228488

[ref70] Watson S. P. Clements M. O. Foster S. J. (1998). Characterization of the starvation-survival response of *Staphylococcus aureus*. J. Bacteriol. 180, 1750–1758. doi: 10.1128/JB.180.7.1750-1758.1998, PMID: 9537371 PMC107086

[ref71] Wilmaerts D. Windels E. M. Verstraeten N. Michiels J. (2019). General mechanisms leading to persister formation and awakening. Trends Genet. 35, 401–411. doi: 10.1016/j.tig.2019.03.007, PMID: 31036343

[ref72] Wood T. K. Song S. (2020). Forming and waking dormant cells: the ppGpp ribosome dimerization persister model. Biofilms 2:100018. doi: 10.1016/j.bioflm.2019.100018, PMID: 33447804 PMC7798447

[ref73] Yin J. Ren W. Huang X. Deng J. Li T. Yin Y. (2018). Potential mechanisms connecting purine metabolism and cancer therapy. Front. Immunol. 9:1697. doi: 10.3389/fimmu.2018.01697, PMID: 30105018 PMC6077182

[ref74] Zhang Y. (2014). Persisters, persistent infections and the Yin-Yang model. Emerg. Microbes. Infect. 3:e3. doi: 10.1038/emi.2014.3, PMID: 26038493 PMC3913823

[ref75] Zhang C. Liang W. Zhang W. Li C. (2016). Characterization of a metalloprotease involved in Vibrio splendidus infection in the sea cucumber, *Apostichopus japonicus*. Microb. Pathog. 101, 96–103. doi: 10.1016/j.micpath.2016.11.005, PMID: 27840223

[ref76] Zhang W. Yamasaki R. Song S. Wood T. K. (2019). Interkingdom signal indole inhibits *Pseudomonas aeruginosa* persister cell waking. J. Appl. Microbiol. 127, 1768–1775. doi: 10.1111/jam.14434, PMID: 31487414

[ref77] Zhou J. Liu L. Shi Z. Du G. Chen J. (2009). ATP in current biotechnology: regulation, applications and perspectives. Biotechnol. Adv. 27, 94–101. doi: 10.1016/j.biotechadv.2008.10.005, PMID: 19026736

[ref78] Zhou W. Yao Y. Scott A. J. Wilder-Romans K. Dresser J. J. Werner C. K.. (2020). Purine metabolism regulates DNA repair and therapy resistance in glioblastoma. Nat. Commun. 11:3811. doi: 10.1038/s41467-020-17512-x, PMID: 32732914 PMC7393131

[ref79] Zou J. Peng B. Qu J. Zheng J. (2022). Are bacterial persisters dormant cells only? Front. Microbiol. 12:708580. doi: 10.3389/fmicb.2021.708580, PMID: 35185807 PMC8847742

