# Supplementary material for: Purine metabolism regulates Vibrio splendidus persistence associated with protein aggresome formation and intracellular tetracycline efflux
Source: Front Microbiol. 2023 Mar 16;14:1127018. doi: 10.3389/fmicb.2023.1127018 (PMC10060992; doi:10.3389/fmicb.2023.1127018)
Supplement: Supplementary file 2 [file Data_Sheet_1.docx]

**SUPPLEMENTARY INFORMATION**


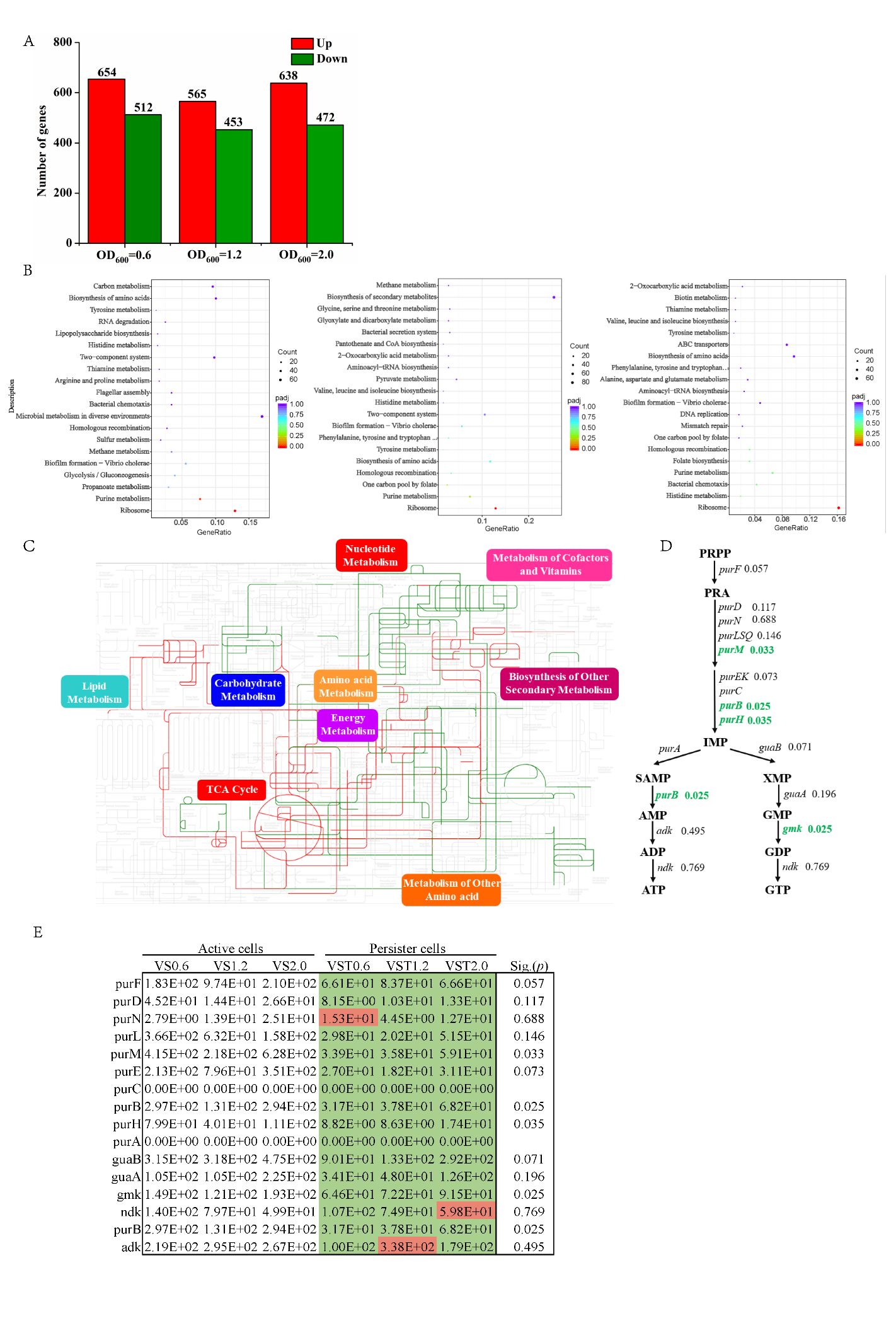


**­**

**Figure S1.** Transcriptomics analysis of tetracycline-induced persister cells (VST) and active cells (VS). (A) Comparison of differentially DEGs among groups VST and VS at OD_600_ = 0.6，VST and VS at OD_600_ = 1.2，VST and VS at OD_600_ = 2.0. Red and green represent up- and down-regulated DEGs, respectively. (B) Top 20 significantly enriched KEGG pathways from DEGs in tetracycline-induced persister cells at OD_600_ = 0.6 (left), OD_600_ = 1.2 (middle) and OD_600_ = 2.0 (right). (C) iPath3.0 (https://pathways.embl.de/) analyzed the enrichment of DGEs in most metabolic pathways. Red and green lines represented the increase and decrease in metabolism respectively. (D) DGEs from purine metabolism displayed significantly difference between VST and VS. The number indicated the significant difference *p* value and green numbers indicated that genes of VST was significantly down-regulation compared with VS. (E) Purine metabolic pathway of transcriptome profiling under VST and VS. The number indicated that gene FPKM value from transcriptome analysis data and the significant difference *p* value. The green boxes mean gene FPKM value was down-regulation; red boxes mean gene FPKM value was up-regulation.


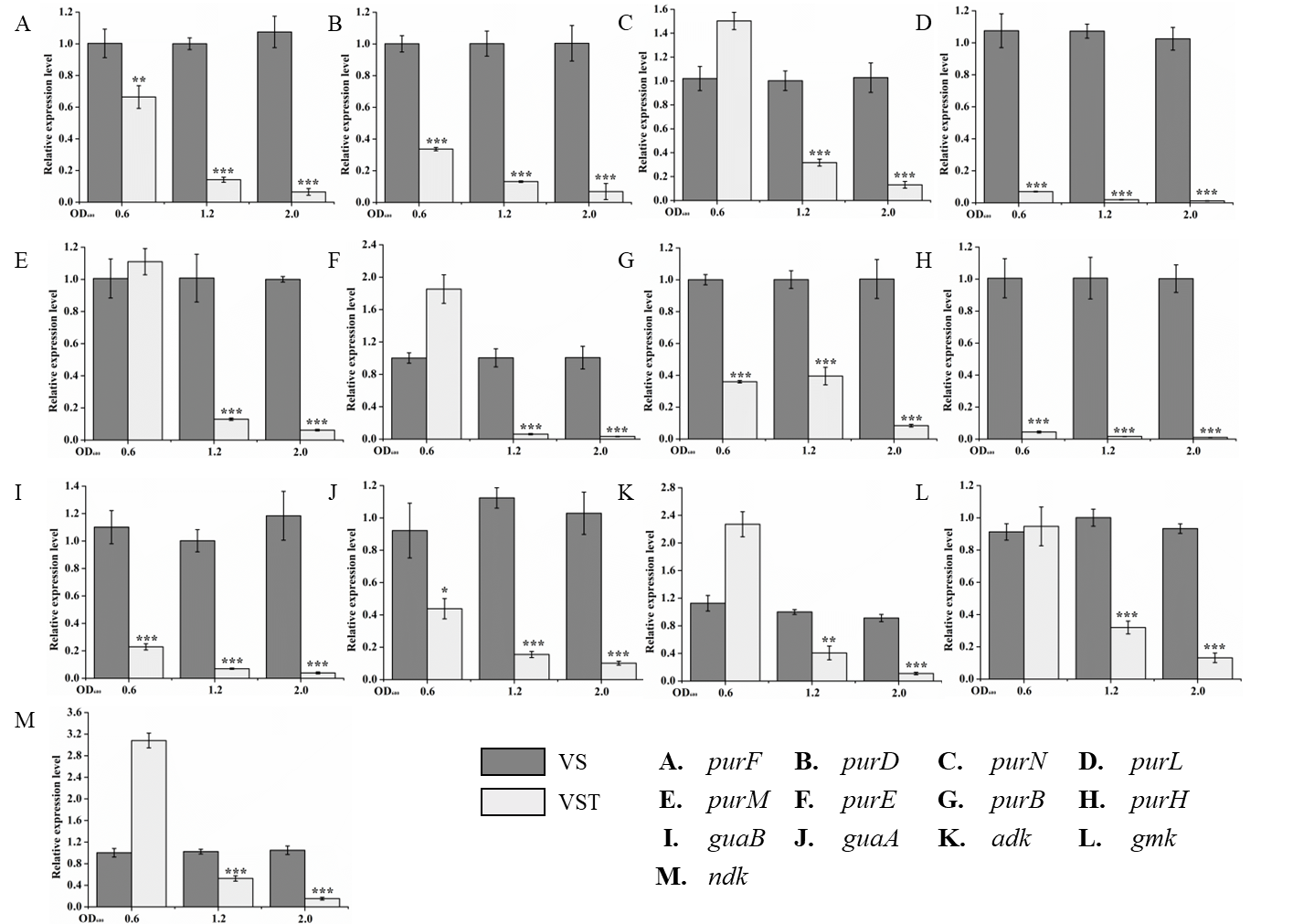


**Figure S2.** The relative mRNA level of purine metabolic pathway in the VS and VST were validated by qRT-PCR. The bars indicated the mean of at least three independent experiments; standard deviation indicated STDEV. (**p* value < 0.05; ***p* value < 0.01; ****p* value < 0.001).


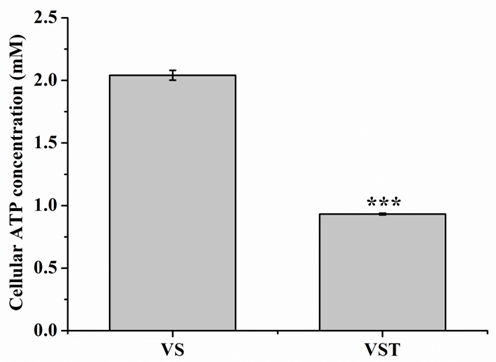


**Figure S3.** Related to Figure 1. Cellular ATP concentration of active cells (VS) and tetracycline-induced persister cells (VST). The bars indicated the mean of at least three independent experiments; standard deviation indicated STDEV. (**p* value < 0.05; ***p* value < 0.01; ****p* value < 0.001).


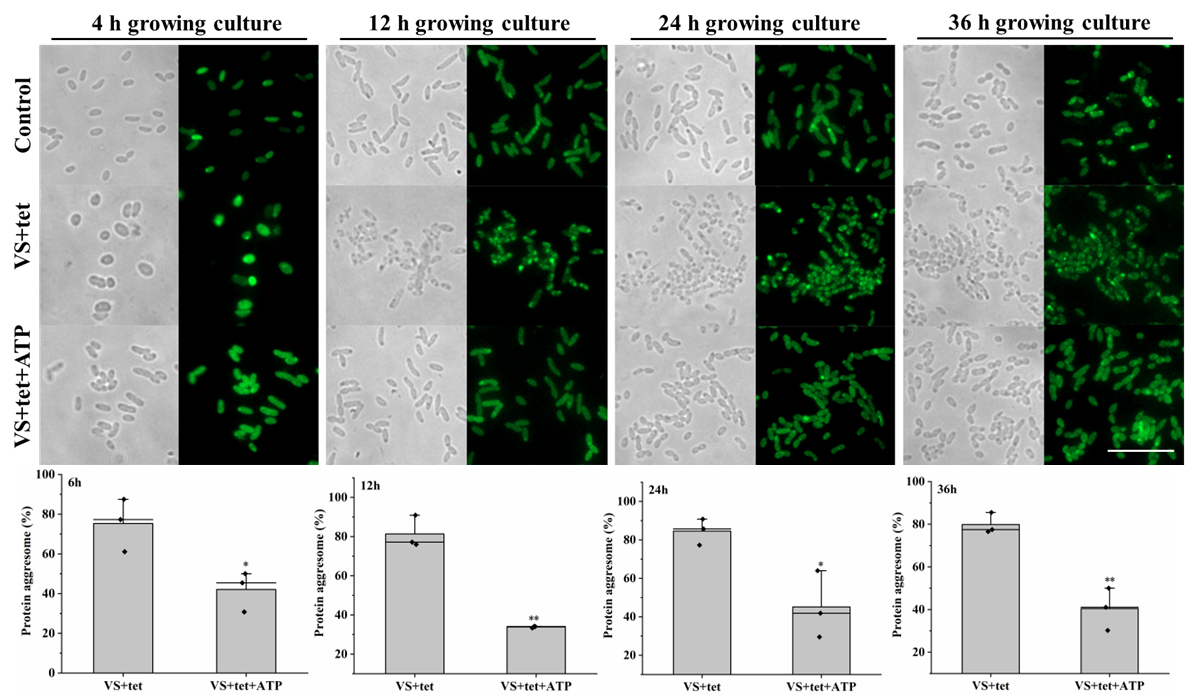


**Figure S4.** Related to Figure 3.

Bright-field and fluorescence images showing that protein aggregates are induced by exogenous 20 mM ATP. And percentage of cells with protein aggresome after different ATP treatment. The bars indicated the mean of percentage of cells with protein aggresome in cell of different treatment at least three independent experiments; standard deviation indicated STDEV. (**p* value < 0.05; ***p* value < 0.01; ****p* value < 0.001). The microscopy settings were the same across all the images.


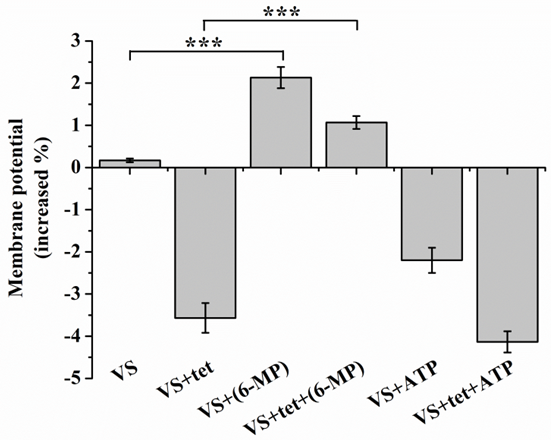


**Figure S5.** Related to Figure 4. Membrane potential of cells were assayed in the presence of 6-MP/ATP.


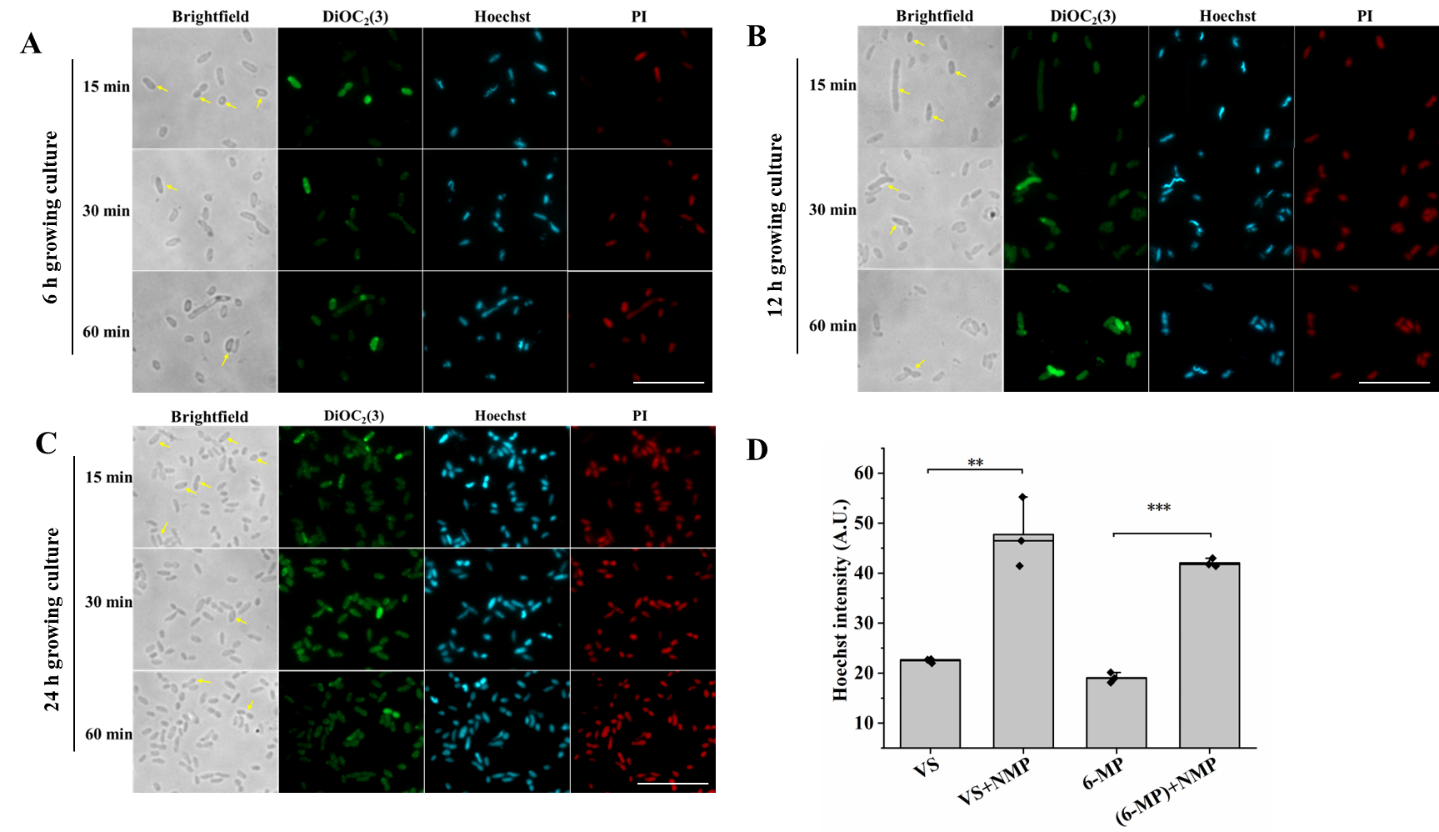


**Figure S6.** Related to Figure 5.

(A), (B) and (C) Bright-field and fluorescence intensity distribution in cells (at three different stages of bacterial growth) are induced by 250 μg/mL tetracycline. Scale bar, 10 μm. (D) Hoechst 33342 fluorescence intensity distribution in cells treated with 6-MP and efflux pump inhibitor NMP. The bars indicated the mean of at least three independent experiments; standard deviation indicated STDEV. (**p* value < 0.05; ***p* value < 0.01; ****p* value < 0.001).


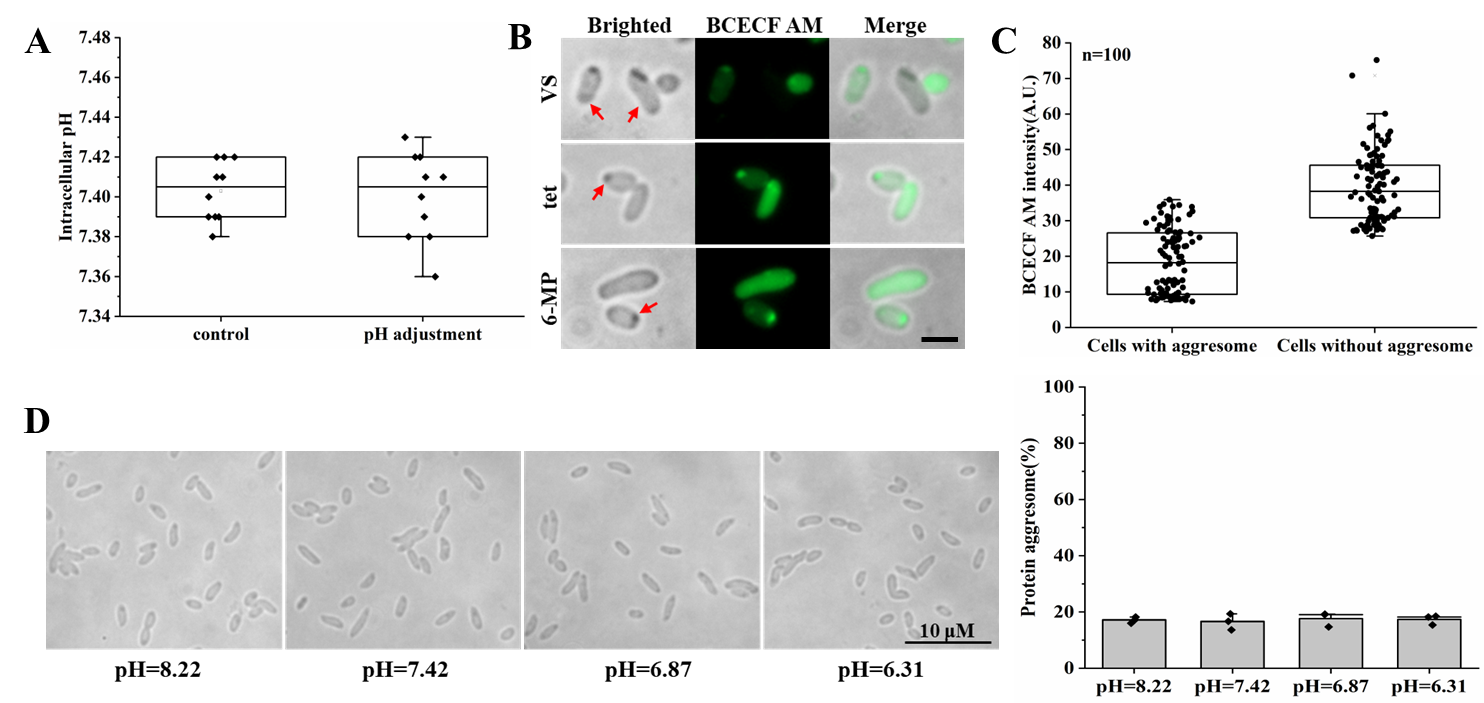


**Figure S7.** Related to Figure 6.

(A) pH of cells after in vivo pH adjustment. In order to see if decreased intracellular pH can induce protein aggresome formation, cells from 12 h culture were treated with or without 40 mM potassium benzoate and 40 mM methylamine hydrochloride for 4 h at 28 ℃. (B) BCECF-AM fluorescence intensity distribution of cells with or without protein aggresomes in cells. Red arrow indicated that the BCECF-AM fluorescence intensity of cells with protein aggresomes was low. (C) BCECF-AM fluorescence intensity of 6-MP-treated cells with or without protein aggresomes. (D) Brightfield images showing that protein aggregates not remained in cells and percentage of cells with protein aggresome after pH adjustment for 4 h. Scale bar, 10 µm.
